# Gendered experiences of providing informal care for older people: a systematic review and thematic synthesis

**DOI:** 10.1186/s12913-021-06736-2

**Published:** 2021-07-23

**Authors:** Ioanna Zygouri, Fiona Cowdell, Avraam Ploumis, Mary Gouva, Stefanos Mantzoukas

**Affiliations:** 1 Department of Medicine, Faculty of Medicine, University of Ioannina, University Campus, P.O. Box: 1186, Zip: 451 10 Ioannina, Greece; 2 School of Nursing and Midwifery, Birmingham City University, Birmingham, UK; 3 Department of Nursing, University of Ioannina, Ioannina, Greece

**Keywords:** Informal carers, Family care, Gender, Qualitative research methods

## Abstract

**Background and purpose:**

The caregiving’s impact on informal carers’ quality of life and gender-based stereotypes make older individuals’ informal care a complex process for which our knowledge is still limited. The purpose of this review is to identify how gender relates to informal carers’ experiences of providing care for people aged 60 years and over with mental and physical health needs by synthesising the available empirical data published between 2000 to 2020.

**Design and methods:**

The systematic method for reviewing and synthesising qualitative data was performed using the PRISMA checklist and ENTREQ statement. The CASP tool was used to examine the quality of the included papers. Thematic synthesis was used as the methodological framework.

**Results:**

This review produced two analytical themes, the impact of gender on the caregivers’ labour and negotiating gender identity with self, society, and cultural norms. While informal caregivers share motivators, a linkage between traditional gender stereotypes impacts caregiving burden and coping strategies. Informal carers’ experiences entail a constant pursuit of self-agency after acquiring the caregiver role. Cultural values and their intersection with gender appear to influence caregivers’ healthy adjustment into their new caregiving identities. The flexibility to move beyond gender boundaries could mediate caregivers’ negotiations between self and society on developing their new caregiving identity. Providing intensive informal primary care to older people affects both men’s and women’s mental and physical health. Gender ideals of the feminine nurturing role further disadvantage women as they determine the caregiving arrangements, the strategies and resources to sustain the caring burden, and the adaptability to experience their new caregiving role positively. Men appear more flexible to debate their hegemonic masculinity and defend their existence in the caregiving role.

**Conclusion and implications:**

Transgressing gender lines and expanding gender possibilities can ease the caregiving burden and strengthen caregivers coping potentials. Health professionals can empower informal careers to challenge gender binaries and expand gender possibilities by intentionally injecting the language of diversity in caring information and caring processes. The review findings outline a path for research on gender identity development in older people’s care.

**Supplementary Information:**

The online version contains supplementary material available at 10.1186/s12913-021-06736-2.

## Background

The ageing of the population has an impact on all aspects of society, including labour, financial markets, family structures and an ever-increasing demand for formal and informal care networks [1]. Informal care is defined as unpaid care provided mainly by family members or other individuals of the patient’s wider social environment at home or care institutions [2]. Informal caregiving, may impact informal carers’ quality of life. Caring for a family member whilst may have positive experiences, including a feeling of gratification, a sense of achievement and a notion of altruism [3]. Nevertheless, a substantial part of the literature suggests a negative impact on their quality of life and wellbeing of informal carers and their ability to mentally and physically manage and cope with the caregiving process [4, 5].

Further research within the context of older people’s care highlights that the intensity of these mental and health effects differs strongly amongst subgroups of caregivers, with female and married caregivers and those providing intensive care experiencing more significant adverse impact [6, 7]. Findings suggest that more stressors and fewer social resources for female caregivers result in lower psychological and physical health than male caregivers [8]. Additionally, cultural gender expectations appear to influence informal caregiving arrangements. Although men are increasingly taking the caregiver’s role, women still appear to constitute the largest proportion of informal caregivers worldwide [1, 9]. The literature attributes this disproportionate involvement of women in caregiving to gender stereotypes that frame caregiving as a “female affair” and consider caregiving as a “feminine type” of activity [10].

Hence, caregiving’s impact on informal caregivers’ quality of life and gender-based stereotypes make older individuals’ informal care a complex, demanding and obscure process for which our knowledge is still limited [11]. Despite the broad research that utilises quantitative primarily methodologies to assess the diverse impacts on caregivers’ physical and psychological well-being, findings are not conclusive on gender differences in caregiving burden. There is an overemphasis on female caregivers, neglecting data on male caregivers [8, 12, 13]. The linkages between gender and the caregiving burden are not explicitly drawn.

Notably, gender studies argue that gender is not an innate characteristic but an accomplishment shaped and influenced by interactions with others, societal power inequalities, and normative social attitudes [14, 15]. Conceptualising gender as an ongoing product of social structures and practices codified and manifested in femininity and masculinity is expected to influence caregiving activities [15–17]. However, caregiving experiences’ potential influence on the continuous process of gender formation is not adequately analysed or discussed in the literature.

Therefore, the purpose of this systematic literature review is to identify how gender relates to informal carers’ experiences of providing care for people aged 60 years and over with mental and physical health needs by synthesising the available empirical qualitative data published between 2000 to 2020. The review question for this systematic literature review is:

- How does gender relate to informal carers’ experiences in older people’s care?

The review question is framed in terms of Population, Exposure, Outcome (PEO) to reflect each of the three examined concepts: ‘Informal Carers’, ‘Care’ and ‘Gender’ [18]. The review question uses the PEO frame as the most appropriate frame to introduce a review question of association/ relationship between two variables, in this case, “care” and “gender” [19]. The objectives of this review are:

- To understand how gender impacts the nature of care provided by informal carers to older individuals.
- To understand how does informal caregiving influence gender identity.

The importance of systematically reviewing the findings of primary empirical qualitative studies on informal caregivers’ caring experiences for older individuals stems from the need to enable a conceptually richer understanding of the gendered experience of being a carer, to address gender inequalities in caring and propose new approaches to research methodologies that account for the complex structures during the whole caregiving trajectory for diverse populations. Synthesis of qualitative data can be invaluable for quantitative research on informal care as it can help identify issues, develop questions for surveys, develop scales, and interpret findings [20]. Informal carers’ mental and physical health is a quintessential component of caring for older people and ensuring good quality of care, and safeguarding their fundamental human rights of living with dignity.

## Design and methods

The systematic method for reviewing and synthesising qualitative data was performed using the Preferred Reporting Items for Systematic Reviews and Meta-Analyses (PRISMA) checklist [21] and the Enhancing Transparency in Reporting the Synthesis of Qualitative research (ENTREQ) statement [22] (see Additional Material 1). This systematic review protocol is registered with the International Prospective Register of Systematic Reviews (PROSPERO). Registration number: CRD42020190576.

### Review methodology

While there are various methodologies for qualitative evidence synthesis, there is an ongoing debate regarding the degree of interpretiveness [22–25]. Qualitative research emerges from different disciplines and traditions with various philosophical underpinnings. Data synthesis methods need to be congruent with the philosophical underpinnings of the primary studies and take extra care not to violate these philosophical assumptions during the synthesis process [23, 26]. For choosing the appropriate data synthesis method, this review applied the RETREAT framework that focuses primarily on qualitative syntheses and guides selecting a suitable synthesis method [23]. Using the framework, Thematic Synthesis methodology was selected (see Table 1 below). Thematic synthesis involves coding included studies to develop descriptive and analytical themes [27]. The specific procedures applied in the thematic synthesis outlined by Thomas and Harden [27] are presented in the following sections.

**Table 1:** Illustrative use of the RETREAT framework for this systematic literature review

Review Question	Qualitative, fixed, descriptive question. Use of the framework Population, Exposure, Outcome (PEO) to formulate the question.
Epistemology	Preference of a method less reliant on epistemological considerations
Time/ Timeframe	Limited, less than a year
Resources	An externally funded project, one author- reviewer with the supervision of three academic staff. Access to the software.
Expertise	PhD researcher, one author/−reviewer, need of an accessible form of synthesis
Audience and Purpose	Part of a doctoral dissertation, academics but also health professionals and practitioners.
Type(s) of Data	An exhaustive search on different databases conducted.
Reporting Standards	ENTREQ: Enhancing transparency in reporting the synthesis of qualitative research
Choice of Method	Thematic Synthesis

### Search strategy

**Fig. 1:**
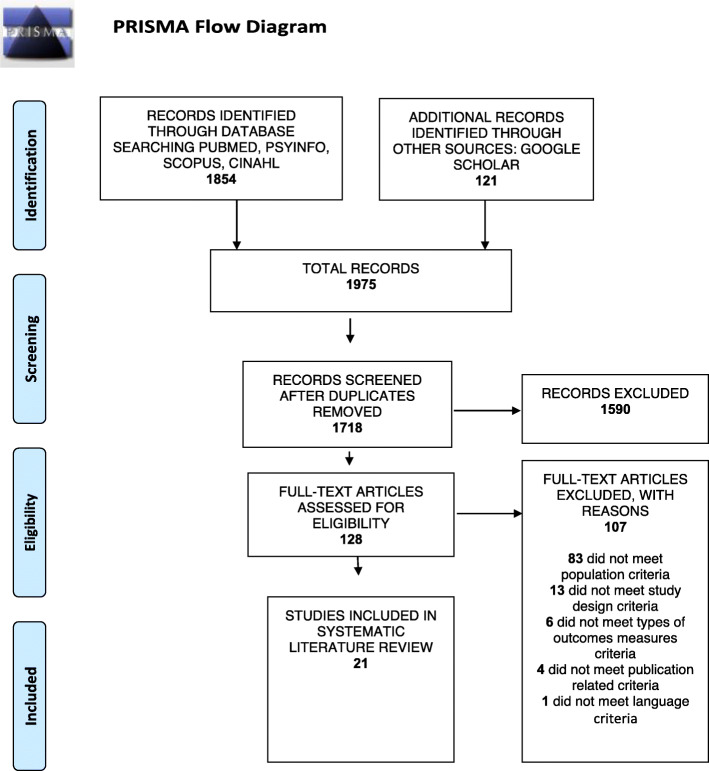
Prisma flow diagram

A comprehensive literature search was conducted in June 2020 in the following databases: PubMed, PsycINFO, Scopus, and Cumulative Index to Nursing and Allied Health Literature. The initial performed search included keywords that reflected the PEO components. In specific, for the “Population”, the keyword used was “informal care”, for “Exposure”, the keyword used was “caregiving”, and for the “Outcome”, the keyword used was “gender”. The types of studies included in the review were qualitative research studies aiming at informal caregivers, emphasising the role of gender in caregiving. The search strategy used both text and index terms, synonyms, and similar terms to correspond to each of the PEO components. Also, the review used Boolean Operators “AND” and “OR” to combine the search terms and truncations to include words variations [28]. An example of a full version of the search strategy is shown in Table 2 (see below). Inclusion and exclusion criteria were set before any search commencement. The eligibility criteria are presented below and summarised in Table 3 [29]. The first author (IZ) independently conducted the screening of studies, and the other members of the authoring team reviewed the screening. Extensive debate and discussion between the first author and other team members helped resolve any discrepancies. The selection process is fully presented in Fig. 1, using the Prisma flow diagram [21].

**Table 2:** Search strategy for PubMed

Population AND	Exposure AND	Outcome AND	Type of Studies
“informal care*” OR “family care*” OR caregiver* OR carer* OR “spousal care*”	caregiving OR eldercare OR gerontolog* OR geriatric* OR ageing OR aging OR aged OR seniors	gender* OR “gender role*” OR “gender norm*” OR “gender relation*” OR “gender identit*” OR “gender continuum” OR feminin* OR masculin* OR “biological sex”	“qualitative research” OR “feminist research” OR phenomenology OR “phenomenological research” OR ethnography OR “action research” OR “grounded theory” OR “ethnographic research” OR “case study research” OR “narrative research” OR “qualitative study”

**Table 3:** Inclusion/ exclusion criteria

	Included	Excluded
Types of studies	Peer-reviewed qualitative studies and qualitative data from mixed methods studies, published between 2000 and 2020, are written in English or Greek language and have a full-text availability.	Studies using quantitative methodologies. Secondary analyses. Grey literature.
Types of participants	Adults, informal primary family caregivers at the time of the interview to older relatives aged 60 years and above with mental and physical health needs.	Participants who spend less than 20 h per week. Participants who provide care at the end of life.
Types of setting	Any residencies where primary caregiving takes place independently of geographical location and cultural context.	Long term care settings, nursing homes, and hospitals where informal caregiving is occasionally taking place.
Outcome measures	Studies focus exclusively on the participants’ caregiving experiences and provide a gender analysis or report outcomes concerning participants’ gender.	Studies report only an assignment of the participants’ sex to specific caregiving tasks and do not perform any further gender analysis or report results relevant to their gender.

### Eligibility criteria

The current review included studies published between 2000 and 2020, written in English or Greek, and the author has full-text availability. Since 2000 gender analysis in research studies has exponentially increased and gender issues acquired an important role in strategy and policymaking; hence this timeframe allowed for the inclusion of a sufficient and appropriate number of studies focusing on gender [30]..

Qualitative studies and qualitative data from mixed methods studies were included as they are best positioned to describe human experiences [31]. Therefore, they served the exploratory nature and focus of this review on expanding the understanding of the gendered informal carers’ experiences [32]. Quantitative studies were excluded as they approach the subject from a statistical and numerical analysis perspective, omitting subjective felt experiences [33]. Secondary analyses were excluded as they reinterpret the original data, depriving the possibility of having a first-hand understanding of the original data [34]. Grey literature was excluded as it lacks peer-reviewing processes and the usual methodological structuring of studies, ergo hampering the potential to filter the text’s relevance, auditing the methodological steps undertaken by the researcher, and the possibility of including undeclared biases or conflicts interests [35].

Participants were adults, informal primary caregivers at the time of the interview to older relatives aged 60 years and over with mental and physical health care needs requiring assistance. This review included only family caregivers [6, 36] to account for the family effect and attachment theories in care. Studies that involved as participants primary family caregivers and their care receivers were included to supplement the data of a caregiving relationship’s dyadic nature [37]. More hours of care are related to more adverse effects on caregivers’ psychological and physical health [38, 39]. The review included only primary caregivers to capture the significant impact of caregiving. Different definitions of primary caregiving exist regarding the time spent on caregiving activities in different countries. Applying different definitions of caregiving, the magnitude of health effects attributable to caregiving can vary substantially [40]. In the USA, primary family caregivers spend approximately 23.7 h per week providing care, particularly those who reside with the care receivers spend approximately 37.4 h or more per week [41]. To be eligible for receiving a caring allowance in the English system, informal carers must provide care more than 35 h per week [42]. Findings show that the impact of care on labour force participation is significant only when individuals provide a high intensity of care (20 h/week or more) and only in the case of co-residential care. This high-intensity caregiving is associated, on average, with a 20% higher prevalence of mental health problems [43]. Based on the above, the threshold for qualifying as the primary caregiver in this review was the provision of care of 20 h/week or more [44]. This threshold followed most of the studies in informal caregiving [44] and allowedfor future studies to compare results [40]. Primary caregivers who cohabitated with the care receivers were included even if the time spent in caregiving was not explicitly mentioned in the study. According to the above, these cohabiting caregivers spend approximately 37.4 h or more per week in care provision. Caregivers who shared caring labour and spent less than 20 h per week in caring activities wereexcluded.

Considering that the physical and mental health comorbidity and the possibility of multiple non diagnosed health issues increase with age, this review involved caregivers of older individuals with various physical and mental health needs [45, 46]. Lastly, participants providing help at the end of life were excluded as they may experience significantly negative emotions not typical in long term caregiving [47].

### Quality assessment

The current review assessed the retrieved articles’ quality using the Critical Appraisal Skills Program qualitative checklist (CASP) [48]. The CASP tool consists of 10 criteria that must be considered when appraising qualitative studies and be answered with a yes, no, or cannot tell. The criteria apply to the following components of the qualitative studies 1) aim, 2) method, 3) design, 4) recruitment, 5) data collection, 6) relationships, 7) ethical issues, 8) analysis, 9) findings, and 10) value of research. Following the guiding principle of transparency in reporting the quality assessment, a summary of each study’s overall quality is presented in the last column of the data extraction Table 4 (see below) regarding the CASP appraisal number [70, 71]. Overall, the studies exhibited high methodological quality fulfilling all or at least eight out of ten criteria. Quality assessment was done independently by the first author (IZ) and reviewed by the other authoring team members. Any discrepancies were resolved by debating them and coming to a final agreement.

**Table 4:** Data extraction, including studies’ details and methodological limitations

Authors, year, Country	Aims of the Study	Study Design, Methodology	Sampling Method and Sample	CASP Tool
Black et al., 2008 USA [49]	To explore experiences of suffering in late life.	Ethnographic research Ethnographic interviews and informal conversations.	Sample was selected from data collected for another funded research project: 4 primary at-home caregivers for wives with dementia, aged 80 and above.	6. Cannot tell. The researchers may have not critically examined their role in the research. 10. No clear suggestions for future research
Cahill, 2000 Australia [50]	To develop an understanding of the caregiving experiences of men looking after spouses diagnosed with dementia.	In depth interviews collected quantitative and qualitative data	Non-probability sample of service users: 26 aged husbands who cared at home for their cognitively impaired wives.	Satisfied all the criteria
Calasanti & Bowen, 2006 USA [51]	To explore the caregiving provided by spouses of persons with Alzheimer’s Disease and related dementias	Qualitative, gender-sensitive, constructivist approach. Semi-structured interviews	Sample recruited from formal agencies, churches, and snowball sampling: 22 primary spousal caregivers for non-institutionalised persons with dementia.	Satisfied all the criteria.
Calasanti & King, 2007 USA [52]	To explore husbands’ experiences of caring for wives with Alzheimer’s disease.	Qualitative, constructivist approach to analyse in-depth interviews	Sample recruited from formal agencies and support groups: 9 caregiving husbands.	7. No reference to ethical considerations
Drummond et al., 2013 Canada [53]	To understand the meaning older women caregivers attribute to their experience of sexuality and intimacy.	Phenomenology approach. Interviews.	Recruitment strategy focused on identifying older caregiving women spouses: 6 community residing women.	6. Cannot tell. The researchers may have not critically examined their role in the research.
Eriksson et al., 2013 Sweden [54]	To explore the gender aspects of long-term caregiving	In depth interview	Participants recruited from an assessment unit at a hospital in South–East Sweden: 12 participants.	Satisfied all the criteria
Flores et al., 2009 USA [55]	To explore the nuances of an ethics of care that constitute caregiving experiences.	Case study. Semi structured qualitative interview	The case study is drawn from a sample of Latina participants in a larger qualitative study: Ana a primary caregiver to her mother.	7. No reference to ethical considerations
Hashizume, 2010 Japan [56]	To explore the experiences of Japanese working women caregivers as they cared for the elderly family member.	Grounded-theory methodology. Open ended interviews around specific topics	Recruitment of women caregivers who met specified criteria: 11 women caregivers including 6 daughters and 5 daughters-in-law.	6. Cannot tell. The researchers may have not critically examined their role in the research.
Hayes et al., 2009 USA [57]	To examine how caregivers of spouses diagnosed with ADRDs perceive identity changes in themselves.	Social constructionist, symbolic interactionist perspective. Intensive interviews.	Spousal caregivers were recruited from support groups: 13 men and 15 women whose spouses had ADRD.	7. Cannot tell. Refers only that the participants agreed to be interviewed
Hayes et al., 2010 USA [58]	To analyse the process of redefining marital relations within the context of couples dealing with Alzheimer’s disease	Intensive qualitative interviewing approach.	Caregivers were selected into the study that met specified criteria: 13 caregiver husbands and 15 caregiver wives.	6. Cannot tell. The researchers may have not critically examined their role in the research.
Hepburn et al., 2002 USA [59]	To identify themes in caregivers’ discourse and reports on patterns among caregivers.	Constant comparative analysis was used to code open-ended interviews	Sample recruited as part of a larger intervention study of family caregivers of community-dwelling persons with dementia: 132 spouses.	Satisfied all the criteria
Holroyd, 2005 China [60]	To address the dilemmas of elderly Chinese women as spousal caregivers in Hong Kong.	In-depth ethnographic approach. Data interpretation via symbolic interactionism.	Convenience sample: 20 elderly wives who were caregivers from Hong Kong.	Satisfied all the criteria
Jones et al., 2002 USA [61]	To describe the process of caring for elderly parents by Asian American women.	Grounded theory methodology. Interviews.	Purposive sample: 41 women (22 Chinese American and 19 Filipino American; aged 38–68 yrs) caring for elderly parents. Subsequent theoretical sample	3. Cannot tell. The researcher did not clearly justify the research design. 6. Cannot tell. The researchers may have not critically examined their role in the research.
Kluczyńska, 2015 Poland [62]	To describe how older men who are caring for their wives construct their masculinity in the face of their new role and tasks.	Semi-structured, in-depth interviews. Thematic analysis coding as a mode of interpretation.	Sample recruited via a local clinic in Poznan: 10 men between 64 and 90 years old who are the primary carers for their wives.	3. Cannot tell. The researcher did not clearly justify the research design. 5. Cannot tell. The researcher did not make the methods explicit, no use of a topic guide.
Kramer, 2005 USA [63]	To illuminate the relationship between gender and burden.	Descriptive qualitative approach and critical poststructuralist feminist approach.	Participants recruited via community care facilities based on specified criteria: 36 adult women caring for highly dependent adults	6. Cannot tell. The researchers may have not critically examined their role in the research.
Mendez-Luck et al., 2008 Mexico [64]	To examine how women in a Mexico City suburb conceptualise the construct of burden.	Phenomenological approach. Semi-structured interviews	Combination of snowball and purposive sampling methods: 41 women.	Satisfied all the criteria
Paillard-Borg & Strömberg, 2014 Japan [65]	To describe the observations and thoughts of one Japanese woman’s experience of living with her elderly parents.	Case study. Open-ended interview was performed and analysed using content analysis.	Case sampling: Miho, a Japanese female caregiver	Satisfied all the criteria
Remennick, 2001 Israel [66]	To explore the experiences of women caregivers with multiple roles.	Qualitative study. Open ended interviews	Women were recruited based on specified criteria: 42 women who lived with the older individual.	6. Cannot tell. The researchers may have not critically examined their role in the research.
Ribeiro et al., 2007 Portugal [67]	To report findings on men’s caregiving experiences.	Semi-structured interviews. Open coding and content analysis	Snowball sampling: 53 elderly men who were caring for chronically ill wives.	Satisfied all the criteria
Silverman, 2013 Canada [68]	To examine the lived reality of women caregivers.	Microethnographic approach. Field research, observations.	Recruitment of caregivers who fit the project’s criteria: 5 caregivers’ dyads.	Satisfied all the criteria
Valadez et al., 2005 USA [69]	To examine Mexican American caregivers’ lived caregiving experiences.	Exploratory study. Semi structured interviews.	Recruitment from Adult Day Care Centers: 15 Mexican American participants.	Satisfied all the criteria

### Data extraction and synthesis

The data extraction and synthesis process commenced with cataloguing the studies details and methodological limitations (see Table 4 below). Consequently, to increase validity and avoid omitting potentially valuable findings for the synthesis, the extracted data were related to the authors’ findings and corresponding participants’ quotations (see Additional Material 2) [71]. Data extracted also concerning the participants’ demographic characteristics (see Additional Material 3). Data extraction and analysis were done independently by the first author (IZ) and reviewed by the other authoring team members.

The three stages of thematic synthesis were applied. In the first stage, the studies’ full text was uploaded to NVivo12 [72] software for qualitative data to make data manageable and inductively read line by line, including the abstract, findings, and discussion sections [27]. The second stage following the inductive coding was subsequent studies coded into pre-existing concepts creating new codes and grouped to create descriptive themes. Finally, in the third stage, the thematic synthesis moves beyond the primary studies and codes to develop conceptual links between codes and descriptive themes and generate a set of analytical themes [27].

## Results

Twenty-one studies met the inclusion criteria and analysed in this review [49–69]. They included primary data representing 484 participants’ views, from which 329 participants were female, and 155 participants were male. Two of the studies [57, 58] included the same male and female participants, and two other [51, 52] studies included the same male participants. Ten of the studies were conducted in the USA, including Native, Asian, African, Hispanic, or Chinese participants; two studies were conducted in Canada, two in Japan, one in Sweden, one in Poland, one in China, one in Mexico, one in Portugal, one in Australia and lastly one in Israel which included Russian women as participants. Participants had different educational and occupational backgrounds, and most cared for a spouse or partner. A smaller number were daughters, daughters in law, a son, and other relatives without the studies defining their relationship status. The informal careers participants’ age ranged from 38 to over 80 years. Twenty studies used qualitative methodology for data collection, and one study used a semi-structured questionnaire designed to collect quantitative and qualitative data. Twelve studies focused on spousal/ partner caregiving, six studies on familial caregiving and three studies included spousal and familial caregiving.

**Fig. 2:**
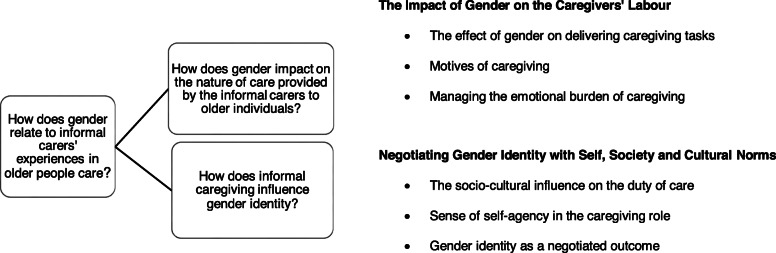
Review question and objectives, descriptive and analytical themes, and the number of corresponded studies

How gender relates to informal carers’ experiences in older individuals’ care manifests in six interconnected descriptive themes grouped into two major analytical themes. The results highlight masculine versus feminine elements that pervade men’s and women’s experiences while caring for older people and emphasise gender disparities. Given the diverse subgroups of caregivers, intersections of gender with the relationship to care receivers that shape different caregiving experiences are highlighted. Figure 2 illustrates the review question and objectives, and the corresponding descriptive themes grouped into two analytical themes. The number of studies representing each of the descriptive themes is also presented in Fig. 2.

### Analytical theme 1: The impact of gender on the caregivers’ labour

The first analytical theme responds to the study’s first objective of understanding how gender impacts the nature of care provided by informal carers to older individuals. It discusses the impact of gender on informal caregivers’ abilities to deliver caregiving tasks, the motives that influence these tasks, and the coping strategies implemented to cope with the caregiving burdens.

#### The effect of gender on delivering caregiving tasks

Men caregivers approached the caregiving process as a new type of occupational role and pragmatically performed this caring role as if executing a set of tasks keeping emotional aspects of care in the background [49–51, 62, 67]. Women, either spouses or daughters, approached the caregiving process with more sentimentality and emotions and anticipated this caring role not as something new but rather as an extension of their existing feminine roles [49–52]. Men in their pragmatic, less emotion-focused, and task-driven approach were more likely to employ enforcement techniques upon care-receivers to comply with the caregiving tasks and prioritise the need to complete tasks instead of responding to emotions [52]. In contrast, women were more reluctant in enforcing compliance as a caregiving technique because they viewed it as contradictory to the perceived nurturing female role. Especially women spouses avoided practices that could diminish their husbands’ sense of self-control [52, 54, 68].

As a result, men felt proud when they successfully performed a caregiving task. In contrast, when they were unsuccessful in performing a task, on the one hand, they felt overwhelmed and, on the other hand, were more forthcoming in asking for professional or familial support as they would have done if performing any task [49–51]. In contrast, women did not display explicit pride or overwhelming sentiments when succeeding (or not) in their caregiving. Instead, they performed caregiving tasks as if they were household tasks without complaining, often underestimating their need for support and de-emphasising the necessity of professional assistance [51, 54, 56]. Finally, men felt more stressed than women in performing caregiving tasks because they considered that the household division of labour was altering, and their role shifted from masculine to more feminine [51].

#### Motives of caregiving

The core motive for both men and women caregivers was the love they felt for their beloved [49, 50, 55, 60, 62–64, 66, 67, 69, 73]. For spousal caregivers, important motivators were their wish to survive as a unit and help their partners sustain a healthy and gendered appearance [49–51, 62]. The duty of care was another common motivator, although its origins differentiated by gender. Women’s sense of duty was primarily rooted in filial obligations, whereas men’s sense of duty was rooted in their feelings of appreciation for their wives and socially imposed imperatives [55, 61, 62, 64, 66]. Gender, monetary restrictions, and ethical resistance to nursing homes were additional reasons women provided care [60, 63, 69]. For men, the belief that their wives would have done the same thing for them, positive memories and commitment to marital vows related to moral values of doing the right thing were additional reasons to provide care [50, 62]. Another significant motivator for men was the positive social visibility they enjoyed as their efforts to perform their caregiving tasks were positively acknowledged by their extended social network [67].

#### Managing the emotional burden of caregiving

Both men and women experienced high emotional burden levels due to their caregiving role. As a result, they expressed feelings of distress and hopelessness, fear for the future, and a sense of loss of the relationship. By deploying their masculine characteristics and adapting and integrating new helpful feminine attributes, men could accept their new role, find purpose in caregiving, and preserve their marriage identity [49, 52]. Moreover, men dealt with the caregiving burden partly by focusing on those rare positive and fulfilling moments that their wives showed clarity of mind reinforcing their perseverance and partly by adapting less favourable tactics, like drinking and self-medicating [49, 52]. By creating reciprocity with their beloved ones and further developing their feminine attributes, women could find meaning in the caregiving role and achieve increased personal growth [60, 61, 64, 65]. Moreover, women dealt with the caregiving burden partly by laughing away its’ consequences and emphasising the selflessness qualities encapsulated in caregiving and partly by suppressing emotions and becoming disinterested in their caregiving tasks. Finally, intersections of gender with ageism stereotypes made adopting successful coping strategies more strenuous for older female spouses in contrast to daughters, who could go against the norm prioritising self-care activities [56, 63, 66, 68].

### Analytical theme 2: Negotiating gender identity with self, society, and cultural norms

The second analytical theme responds to the second objective of this study of understanding how informal caregiving influences gender identity. It discusses the sociocultural influences on informal caregivers, the role of self-agency in caregiving, and caregivers’ negotiated gender identity. This theme reveals the diversity of caregiving effects by interconnecting gender, cultural and societal influences that eventually (re) shape the caregivers’ identity in older people’s care.

#### The sociocultural influence on the duty of care

Social norms shaped by governmental influences, religious systems, cultural principles, and societal ethics define women as the primary caregiver, fulfilling family values, moral considerations, and gender expectations [55, 62, 64, 69]. These social norms imprint on female caregivers’ stereotypical gender traits such as affection, dutifulness, and compliance when carrying out caregiving activities [63, 68, 69]. In specific, wives were expected to provide informal care to their husbands in their older life and daughters were held to a distinct set of ethical standards higher than sons that were excused from caregiving obligations. For men spouses, social norms’ influence was based on the sense of duty as this emerged from their religious marital vows and their faith in God [49, 50]. By remaining loyal to their vows, men complied with cultural expectations, personal beliefs, and positive social visibility [67]. Transgression of caregiving expectations defined from social norms bestows a greater sense of guilt for women. In contrast, men feel lower levels of shame when they fail in their caregiving responsibilities and require institutional care [56, 60, 61, 65].

#### Sense of self-agency in the caregiving

Becoming a caregiver signified a life turn and a rupture in the planned continuity of caregivers’ lifespan, inevitably creating a loss of control and self-agency. Men often felt trapped in their spouses’ illness and alienated from their life ambitions [49, 50]. Restoration of continuity of men’s life and regaining control of life events required the retrieval of the masculine trait of becoming a defender and undertaking responsibility for salvaging their marriage identity [49, 50, 62]. Maintaining a sense of self-agency appeared a more strenuous task for women. Women felt socially restricted in pursuing their interests, personal needs and career ambitions, as well as the loss of their sexual identity and social status [53, 54, 60, 65, 66]. Especially older women felt greater restriction levels than daughters due to the intersection of gender with age stereotypes. The primary way to deal with these restrictions was for women to focus on the self rather than the care receiver’s needs by cultivating personal growth and attempting to become as detached as possible from the care receiver. Overall, coming to terms and accepting the new caregiving role was crucial for both men and women for achieving a sense of wellbeing and hopefulness [54–56, 59, 61, 65].

#### Gender identity as a negotiated outcome

The core element in accepting the caregivers’ role and managing the caregiving burden is to (re) negotiate traditional gender identity features. Men and women caregivers were willing to or had already materialised the crossing of gender lines while performing their caregiving roles [51]. Men who were willing to express feelings and emotions clearly and inclined to redefine the traditional masculine identity to include more feminine traits felt greater inner peacefulness in executing their caregiving duties [49, 52, 67]. Similarly, women who were willing to become more pragmatic and task-focused in their caregiving found the necessary space to express their interests, sexuality, personal needs and career ambitions [53, 57, 58, 63]. Finally, primarily women but also in some cases men as well, found this crossing of gender lines not easy and considered it a risky endeavour out of fear of endangering their sense of social belongingness. An intersection of care to the relationship to care receiver appeared to disadvantage more older wives than daughters on negotiating gender boundaries, as their gender and sexual identity were closely interwoven to their husbands [53, 58, 60, 63].

## Discussion

This review performed a thematic synthesis of qualitative studies on how gender relates to informal carers’ experiences in older people’s care. Two analytical themes emerged from data synthesis: a) the impact of gender on the caregivers’ labour, and b) negotiating gender identity with self, society, and cultural norms. These analytical themes reveal how important gender is to the experience of caring for older people. The results show that gender determines the caregiving arrangements within the households; gender imbues the caregivers’ motives to provide care, it affects how they deliver the caring tasks and their coping strategies to mediate the caregiving burden. Gender has significant implications on how flexible individuals adjust to their new caregiving identity at the crucial and abrupt moment that they become carers and their life changes and through the whole course of care. Gender intersects with the relationship to the care receivers and other identity-defining characteristics as age and ethnicity to further disadvantage subgroups of caregivers. All these findings together shape a significant gender impact in the informal family provision of care to older people.

The current review asserts that women caregivers influenced by traditional feminine roles are much more emotionally involved in caregiving. In contrast, male caregivers influenced by traditional masculine roles are more detached and task-oriented in their caregiving [17, 74]. Prescriptive components of gender stereotypes that construct the beliefs about what men and women should do suggest that women are supposed to be warm, sensitive, cooperative and avoid dominance. In contrast, men are supposed to be agentic, assertive, competitive, independent and avoid weakness [75]. These prescriptive components of gender stereotypes in part explain this review’s finding of men’s tendency to reinforce compliance by providing care in a managerial manner, placing the practical completion of tasks above the means used to achieve it.

Following previous reviews, affection, reciprocity, feelings of compassion, and the duty to provide care appeared as common motivators for both women and men [13, 76]. Moreover, this review highlighted the linkage of these motivators to cultural imperatives of the dutiful spouse, husband, daughter, son image that preserves social and family harmony [77]. However, these motivators were more potent for men as they added to men’s masculine identity, offering them further social recognition. While significant for women, these motivators lacked the potency they had for men because they merely upheld the nurturing feminine identity without adding any further credit other than that related to their existing recognition of their gender role.

In agreement with previous studies that used quantitative and qualitative methodologies,women appeared to express a higher burden than men [6, 7]. Nevertheless, men also suffered from the caregiving burden, but they were less likely to share their negative feelings emanating from this burden and more willing to ask for support [13, 78]. The literature associates higher burden levels with emotional-focused coping strategies primarily used by women, whereas lower burden levels with problem-solving approaches used primarily by men [79]. A set of studies report a similar linkage between caregiving burden and coping strategies concluding that men and women implementing an emotional-focused coping strategy tend to display greater burden levels [17, 80]. This review expands on the linkage between caregiving burden levels and coping strategies, suggesting that caregivers expressed a lesser burden level when applying a combination of emotional and problem-focused coping strategies.

Concerning the intersection of gender and relationship in caregiving burden, findings from this review concur with most quantitative studies. Previous findings showed that wives and daughters report similar stress and depressive symptoms. The depressive symptoms were more significant and induced greater vulnerability for wives’ self-esteem than did for daughters [81, 82]. Similarly, this review concluded that both wives and daughters expressed a high level of burden when they felt trapped in the caregiving obligations. However, that was more prominent for older wives as it further intersected with age-related restrictions creating an environment of limited resources for these women to preserve a sense of agency and positive self-image. Studies that identified higher quality of formal and informal support to caregivers concluded that the perceived burden for caregivers is lower, irrelevant of gender, relationship or age [83–86]. This review also demonstrates that the intensity of caregiving and the lack of support lead to greater levels of burden of both genders and all ages.

Sociocultural factors further disadvantaged women’s healthy adjustment in the new caregiver role. Previous literature suggests that both ethnicity and gender impact caregiving arrangements as differences in the construction of gender across countries strengthen the normative gender ideals on how people behave and explain their actions [20, 87, 88]. Accordingly, the findings of this review show that social imperatives and religious beliefs impact the female coping potentials in dealing with the caregiving burden. This is more evident in non-Western settings that are either influenced by religious paradigms such as Catholicism and Confucianism or by traditional submissive female roles that approach care as a form of purification. The fact that women conceive caregiving as a normative part of the family life implies that cultural values may not directly render caregiving burdensome but may impact the choices and the use of coping strategies that eventually prohibit women from seeking help and interventions [77, 89–92].

Furthermore, this review asserts that identity development is a contextualised phenomenon characterised by dynamic interactions between individuals and societies. More importantly, the sense of identity continuation across time and situations leads to a sense of well-being and confirms the individual’s self-agency experiences [93, 94]. The participants in this review on becoming caregivers experienced an abrupt change of role, which forced a shift in their identity. The more successful individuals were in adapting to the new identity role as caregivers, the greater the potential to increase their self-agency and sense of control and their overall sense of well-being. Gender influenced men and women differently in adapting to the new caregiving identity. Women felt physically and emotionally exhausted from the new caregiving role because it emphasised and magnified traditional female stereotypes of family caretakers, further restricting them from pursuing their interests, personal needs, career ambitions, sexual identity and social status. In contrast, men caregivers were able to block some of the emotional aspects of care by suppressing emotions and re-patterning the caregiver role into a challenge that, when successfully achieved, provided a sense of honour in the success and a sense of self-agency [95]. Nevertheless, this appeared more of a short-term solution for men, and in the long term, the suppressed emotions added to the caregiving burden [95].

This review concludes that while caring for older people, both men and women can ease the caregiving burden and strengthen their coping strategies by transgressing gender lines. Women who become more pragmatic and task-focused and men who express feelings and emotions can move beyond the socially constructed gender boundaries, attaining greater peace with the caregiving process. The literature defines this transgression of gender boundaries as psychological flexibility that can adapt to contextual changes and situational demands, shifting mindsets or behavioural repertoires [96, 97]. This final point is vital for health professionals and formal carers in successfully supporting informal carers to adapt to their new role. Healthcare professionals can empower informal carers to challenge the rigid gender binary in informal caring by developing educational programs and communication patterns that expand gender possibilities by intentionally injecting the language of diversity and inclusivity in the caring process [98, 99].

### Limitations

This review is limited to existing data available in the literature, and therefore, other variables for interpreting data as nuances and context were not available in answering the review question and analysing the data [100]. Potential author biases related to the primary studies included in the review and possible influences in these primary studies’ research process may impact the review’s conclusions. Also, the imbalances concerning gender distribution among caregivers in the reviewed studies, given that most of the studies included female participants, may influence the review’s outcomes. The study participants were all primary family caregivers who spend 20 h per week caring for older individuals. Therefore, transferability of the results to other populations of carers who spend less intensive time in caring activities may not be applicable. In addition, it should be noted that data for male participants in this study were derived mainly from spousal caregivers as only one study included one male participant who cared for an older parent. More research exploring the experiences of sons as caregivers is needed. This review explored informal primary family carers’ experiences for older people with various health needs and independently of race, ethnicity, gender, socioeconomic status, and geographic location, thus creating a non-homogeneous review sample that may impact the review’s applicability findings in specific contexts. Also, non peer-reviewed work was not included in this review. Finally, the disproportionate emphasis of the literature on dementia-related diseases may have resulted in an imbalance in caregiving needs and caregiving expectations. Hence, future studies should be more forthcoming in studying caregivers’ experiences of older individuals with other health conditions.

## Conclusion and implications

This systematic literature review aimed to understand how gender relates to older people’s informal carers’ experiences. Providing intensive informal primary care to older people affects both men’s and women’s mental and physical health. Gender stereotypes of the feminine nurturing role further disadvantage women as they determine caregiving arrangements, the strategies and resources available to sustain the caring burden, and the adaptability to experience their new caregiving role positively. Men appear more flexible to debate their hegemonic masculinity and defend their existence in the caregiving role. The common motivators for both women and men informal careers are their affection, feelings of compassion, and the duty to provide care for their beloved ones. While women and men, informal caregivers share motivators, traditional gender stereotypes influence informal women and men caregivers differently. Women caregivers influenced by traditional feminine roles are more emotionally involved, whereas men influenced by traditional masculine roles are more practical in completing caregiving tasks. Furthermore, a linkage between traditional gender stereotypes impacts women’s and men’s felt caregiving burden and coping strategies employed to deal with it. Gender stereotypes influence men and women differently in adapting to the new caregiving identity. Women implement more emotional-focused coping strategy, whereas men implement more problem-focused coping strategies. Transgressing gender lines and expanding gender possibilities can ease the caregiving burden and strengthen caregivers coping potentials. Health professionals can empower informal careers to challenge gender binaries and expand gender possibilities by intentionally injecting the language of diversity in caring information and caring processes. Finally, the review findings outline a path for research on gender identity development in older people’s care, emphasising the intersection of gender with other identity-defining characteristics as ethnicity, age and class. There is a need for gender-sensitive and culturally informed multimethod research that involves participants across the gender continuum. Future studies need to move beyond typical femininity and masculinity assessments while exploring informal carers’ gendered experiences.

## Supplementary Information

**Additional file 1.**

**Additional file 2.**

**Additional file 3.**

## Data Availability

All data analysed during this study are included in this published article (and its additional files).

## Abbreviations

PRISMA: Preferred reporting items for systematic review and meta-analysis
ENTREQ: Enhancing transparency in reporting the synthesis of qualitative research
CASP: Critical Appraisal Skills Programme

## References

[1] WHO (2020). Decade of healthy ageing: Baseline report.

[2] Skinner MS, Lorentzen H, Tingvold L, Sortland O, Andfossen NB, Jegermalm M. Volunteers and informal caregivers’ contributions and collaboration with formal caregivers in Norwegian long-term care. J Aging Soc Policy. 2020:1–26. Available from: 10.1080/08959420.2020.1745988. Cited 2021 Jan 9. 10.1080/08959420.2020.1745988 32252614

[3] MacKenzie A, Greenwood N (2012). Positive experiences of caregiving in stroke: A systematic review. Disabil Rehabil.

[4] Mosquera I, Vergara I, Larrañaga I, Machón M, del Río M, Calderón C (2016). Measuring the impact of informal elderly caregiving: a systematic review of tools. Qual Life Res.

[5] Bauer JM, Sousa-Poza A (2015). Impacts of informal caregiving on caregiver employment, health, and family. J Popul Ageing.

[6] Bom J, Bakx P, Schut F, Van Doorslaer E (2019). The impact of informal caregiving for older adults on the health of various types of caregivers: a systematic review. Gerontologist.

[7] Xiong C, Biscardi M, Astell A, Nalder E, Cameron JI, Mihailidis A (2020). Sex and gender differences in caregiving burden experienced by family caregivers of persons with dementia: A systematic review. PLoS One.

[8] Pinquart M, Sörensen S. Gender differences in caregiver stressors, social resources, and health: An updated meta-analysis. J Gerontol B Psychol Sci Soc Sci. 2006. p. 61. Available from: https://pubmed.ncbi.nlm.nih.gov/16399940/. Cited 2021 Feb 10. 10.1093/geronb/61.1.p33 16399940

[9] Calvó-Perxas L, Vilalta-Franch J, Litwin H, Turró-Garriga O, Mira P, Garre-Olmo J. What seems to matter in public policy and the health of informal caregivers? A crosssectional study in 12 European countries. PLoS One. 2018;13(3). Available from: https://pubmed.ncbi.nlm.nih.gov/29518147/. Cited 2021 Jan 9. 10.1371/journal.pone.0194232 PMC5843287 29518147

[10] Cunha V, Atalaia S (2018). The gender (ed) division of labour in Europe: patterns of practices in 18 EU countries. Sociol Probl e Práticas.

[11] Harvath TA, Mongoven JM, Bidwell JT, Cothran FA, Sexson KE, Mason DJ (2020). Research priorities in family caregiving: process and outcomes of a conference on family-centered care across the trajectory of serious illness. Gerontologist.

[12] Macdonald M, Martin-Misener R, Weeks L, Helwig M, Moody E, Maclean H (2020). Experiences and perceptions of spousal/partner caregivers providing care for community-dwelling adults with dementia: A qualitative systematic review. JBI Evid Synthesis.

[13] Sharma N, Chakrabarti S, Grover S (2016). Gender differences in caregiving among family - caregivers of people with mental illnesses. World J Psychiatry.

[14] West C, Zimmerman DH (1987). Doing gender. Gend Soc.

[15] West C, Fenstermaker S (1995). Doing difference. Gend Soc.

[16] Weldon SL (2007). Difference and social structure: Iris young’s critical social theory of gender. Constellations.

[17] Calasanti T (2010). Gender relations and applied research on aging. Gerontologist.

[18] Bettany-Saltikov J (2010). Learning how to undertake a systematic review: part 1. Nurs Stand.

[19] Moola S, Munn Z, Sears K, Sfetcu R, Currie M, Lisy K (2015). Conducting systematic reviews of association (etiology): The Joanna Briggs Institute’s approach. Int J Evid Based Healthc.

[20] Dilworth-Anderson P, Williams IC, Gibson BE (2002). Issues of race, ethnicity, and culture in caregiving research: a 20-year review (1980–2000). Gerontologist.

[21] Moher D, Liberati A, Tetzlaff J, Altman DG (2009). Preferred reporting items for systematic reviews and meta-analyses: the PRISMA statement. PLoS Med.

[22] Tong A, Flemming K, McInnes E, Oliver S, Craig J (2012). Enhancing transparency in reporting the synthesis of qualitative research: ENTREQ. BMC Med Res Methodol.

[23] Booth A, Noyes J, Flemming K, Gerhardus A, Wahlster P, van der Wilt GJ (2018). Structured methodology review identified seven (RETREAT) criteria for selecting qualitative evidence synthesis approaches. J Clin Epidemiol.

[24] Doyle LH (2003). Synthesis through meta-ethnography: paradoxes, enhancements, and possibilities. Qual Res.

[25] Kastner M, Tricco AC, Soobiah C, Lillie E, Perrier L, Horsley T (2012). What is the most appropriate knowledge synthesis method to conduct a review? Protocol for a scoping review. BMC Med Res Methodol.

[26] Peters M, Godfrey C, McInerney P, Munn Z, Trico A, Khalil H. Chapter 11: scoping reviews. In: Aromataris E, Munn Z, editors. JBI Manual for Evidence Synthesis, JBI; 2020. Available from https://synthesismanual.jbi.global. 10.46658/JBIMES-20-12.

[27] Thomas J, Harden A (2008). BMC Med Res Methodol.

[28] F. Etten-Jamaludin van, Deurenberg HWJ. A practical guide to PubMed : the guide which helps you to search quickly and efficiently in PubMed. Springer; 2009. Available from: https://research.tue.nl/en/publications/a-practical-guide-to-pubmed-the-guide-which-helps-you-to-search-q. Cited 2021 Jan 9

[29] Needleman IG (2002). A guide to systematic reviews. J Clin Periodontol.

[30] Clayton JA, Tannenbaum C (2016). Reporting sex, gender, or both in clinical research?. JAMA.

[31] de Vet HCW, Verhagen AP, Logghe I, Ostelo RWJG (2005). Literature research: aims and design of systematic reviews. Aust J Physiother.

[32] Ring N, Ritchie K, Mandava L, Jepson R. A guide to synthesising qualitative research for researchers undertaking health technology assessments and systematic reviews. NHS Qual Improv Scotland (NHS QIS). 2011. Available from: www.nhshealthquality.org. Cited 2021 Jan 9.

[33] Pearson M (2008). Synthesizing qualitative and quantitative health evidence: a guide to methods*.* - by Pope, C., Mays, N., and Popay, J. Sociol Health Illn.

[34] Erwin EJ, Brotherson MJ, Summers JA (2011). Understanding qualitative metasynthesis. J Early Interv.

[35] Benzies KM, Premji S, Hayden KA, Serrett K (2006). State-of-the-evidence reviews: Advantages and challenges of including grey literature. Worldviews Evid-Based Nurs.

[36] Karantzas G, Simpson JA (2015). Attachment and aged care. Attach Theory Res New Dir Emerg Themes.

[37] Lyons KS, Zarit SH, Sayer AG, Whitlatch CJ (2002). Caregiving as a dyadic process: perspectives from caregiver and receiver. J Gerontol Ser B Psychol Sci Soc Sci.

[38] Pinquart M, Sörensen S (2003). Differences between caregivers and noncaregivers in psychological health and physical health: A meta-analysis. Psychol Aging.

[39] Kim H, Chang M, Rose K, Kim S (2012). Predictors of caregiver burden in caregivers of individuals with dementia. J Adv Nurs.

[40] Schulz R, Newsom J, Mittelmark M, Burton L, Hirsch C, Jackson S (1997). Health effects of caregiving: The caregiver health effects study: An ancillary study of the cardiovascular health study. Ann Behav Med.

[41] Public Policy Institute A (2019). Caregiving in the U.S. 2020 - AARP Research Report.

[42] What is Carer’s Allowance? - Carers UK. Available from: https://www.carersuk.org/about-us/71-wales/. Cited 2021 Apr 26.

[43] Rodrigues R, Schulmann K, Schmidt A, Kalavrezou N, Matsaganis M. The indirect costs of long-term care. 2013; Available from: https://www.euro.centre.org/publications/detail/415. Cited 2021 Apr 28.

[44] Informal care in Europe - Publications Office of the EU. Available from: https://op.europa.eu/en/publication-detail/-/publication/96d27995-6dee-11e8-9483-01aa75ed71a1. Cited 2021 Apr 26.

[45] Karlamangla A, Tinetti M, Guralnik J, Studenski S, Wetle T, Reuben D. Comorbidity in older adults: nosology of impairment, diseases, and conditions. J Gerontol Ser A Biol Sci Med Sci. 2007;62(3):296–300. Available from: 10.1093/gerona/62.3.296. Cited 2021 Jan 9. 10.1093/gerona/62.3.296 17389727

[46] Lorem GF, Schirmer H, Wang CEA, Emaus N. Ageing and mental health: Changes in self-reported health due to physical illness and mental health status with consecutive cross-sectional analyses. BMJ Open. 2017;7(1). Available from: 10.1136/bmjopen-2016-013629. Cited 2021 Jan 9. 10.1136/bmjopen-2016-013629 PMC5253588 28100564

[47] Funk L, Stajduhar KI, Toye C, Aoun S, Grande GE, Todd CJ (2010). Part 2: Home-based family caregiving at the end of life: A comprehensive review of published qualitative research (1998–2008). Palliat Med.

[48] Critical Appraisal Skills Programme (2019). CASP (Qualitative) Checklist.

[49] Black HK, Schwartz AJ, Caruso CJ, Hannum SM (2008). How personal control mediates suffering: elderly husbands’ narratives of caregiving. J Mens Stud.

[50] Cahill S (2000). Elderly husbands caring at home for wives diagnosed with Alzheimer’s disease: are male caregivers really different?. Aust J Soc Issues.

[51] Calasanti T, Bowen ME (2006). Spousal caregiving and crossing gender boundaries: maintaining gendered identities. J Aging Stud.

[52] Calasanti T, King N (2007). Taking ‘Women’s Work’ ‘Like a Man’: Husbands’ experiences of care work. Gerontologist.

[53] Drummond JD, Brotman S, Silverman M, Sussman T, Orzeck P, Barylak L (2013). The impact of caregiving. Affilia.

[54] Eriksson H, Sandberg J, Hellström I (2013). Experiences of long-term home care as an informal caregiver to a spouse: Gendered meanings in everyday life for female carers. Int J Older People Nursing.

[55] Flores YG, Hinton L, Barker JC, Franz CE, Velasquez A (2009). Beyond familism: A case study of the ethics of care of a latina caregiver of an elderly parent with dementia. Health Care Women Int.

[56] Hashizume Y (2010). Releasing from the oppression: Caregiving for the elderly parents of Japanese working women. Qual Health Res.

[57] Hayes J, Boylstein C, Zimmerman MK (2009). Living and loving with dementia: negotiating spousal and caregiver identity through narrative. J Aging Stud.

[58] Hayes J, Zimmerman MK, Boylstein C (2010). Responding to symptoms of Alzheimer’s disease: Husbands, wives, and the gendered dynamics of recognition and disclosure. Qual Health Res.

[59] Hepburn K, Lewis ML, Narayan S, Tomatore JB, Bremer KL, Sherman CW (2002). Discourse-derived perspectives: Differentiating among spouses’ experiences of caregiving. Am J Alzheimers Dis Other Dement.

[60] Holroyd E (2005). Developing a cultural model of caregiving obligations for elderly Chinese wives. West J Nurs Res.

[61] Jones PS, Zhang XE, Jaceldo-Siegl K, Meleis AI (2002). Caregiving between two cultures: An integrative experience. J Transcult Nurs.

[62] Kluczyńska U (2015). Older husbands as carers - constructions of masculinity in context of care-giving. Stud Humanist AGH.

[63] Kramer MK (2005). Self-characterizations of adult female informal caregivers: gender identity and the bearing of burden. Res Theory Nurs Pract.

[64] Mendez-Luck CA, Kennedy DP, Wallace SP (2008). Concepts of burden in giving care to older relatives: A study of female caregivers in a Mexico City neighborhood. J Cross Cult Gerontol.

[65] Paillard-Borg S, Strömberg L (2014). The importance of reciprocity for female caregivers in a super-aged society: a qualitative journalistic approach. Health Care Women Int.

[66] Remennick LI (2001). “All my life is one big nursing home”: Russian immigrant women in Israel speak about double caregiver stress. Womens Stud Int Forum.

[67] Ribeiro O, Paúl C, Nogueira C (2007). Real men, real husbands: caregiving and masculinities in later life. J Aging Stud.

[68] Silverman M (2013). Sighs, smiles, and worried glances: How the body reveals women caregivers' lived experiences of care to older adults. J Aging Stud.

[69] Valadez AA, Lumadue C, Gutierrez B, de Vries-Kell S (2005). Family caregivers of impoverished mexican american elderly women: the perceived impact of adult day care centers. Fam Soc J Contemp Soc Serv.

[70] Majid U, Vanstone M (2018). Appraising qualitative research for evidence syntheses: a compendium of quality appraisal tools. Qual Health Res.

[71] Noyes J, Booth A, Flemming K, Garside R, Harden A, Lewin S (2018). Cochrane qualitative and implementation methods group guidance series—paper 3: methods for assessing methodological limitations, data extraction and synthesis, and confidence in synthesized qualitative findings. J Clin Epidemiol.

[72] from: https://www.qsrinternational.com/nvivo-qualitative-data-analysis-software/home. Cited 2021 Jan 23.

[73] Mazure CM, Jones DP. Twenty years and still counting: Including women as participants and studying sex and gender in biomedical research. BMC Womens Health. 2015;15(1). Available from: https://pubmed.ncbi.nlm.nih.gov/26503700/. Cited 2021 Jan 9. 10.1186/s12905-015-0251-9 PMC4624369 26503700

[74] Hong SC, Coogle CL (2016). Spousal caregiving for partners with dementia: a deductive literature review testing calasantis gendered view of care work. J Appl Gerontol.

[75] Koenig AM (2018). Comparing prescriptive and descriptive gender stereotypes about children, adults, and the elderly. Front Psychol.

[76] Morgan T, Ann Williams L, Trussardi G, Gott M (2016). Gender and family caregiving at the end-of-life in the context of old age: A systematic review. Palliat Med.

[77] Pharr JR, Dodge Francis C, Terry C, Clark MC (2014). Culture, caregiving, and health: exploring the influence of culture on family caregiver experiences. ISRN Public Health.

[78] Robinson CA, Bottorff JL, Pesut B, Oliffe JL, Tomlinson J (2014). The male face of caregiving: a scoping review of men caring for a person with dementia. Am J Mens Health.

[79] Papastavrou E, Kalokerinou A, Papacostas SS, Tsangari H, Sourtzi P (2007). Caring for a relative with dementia: Family caregiver burden. J Adv Nurs.

[80] Baker KL, Robertson N (2008). Coping with caring for someone with dementia: Reviewing the literature about men. Aging Ment Health.

[81] Chappell NL, Dujela C, Smith A (2015). Caregiver well-being: intersections of relationship and gender. Res Aging.

[82] Simpson C, Carter P (2013). Mastery a comparison of wife and daughter caregivers of a person with dementia. J Holist Nurs.

[83] Savundranayagam MY, Montgomery RJV, Kosloski K (2011). A dimensional analysis of caregiver burden among spouses and adult children. Gerontologist.

[84] Juntunen K, Salminen AL, Törmäkangas T, Tillman P, Leinonen K, Nikander R (2018). Perceived burden among spouse, adult child, and parent caregivers. J Adv Nurs.

[85] Galvin JE, Duda JE, Kaufer DI, Lippa CF, Taylor A, Zarit SH (2010). Lewy body dementia: Caregiver burden and unmet needs. Alzheimer Dis Assoc Disord.

[86] Zarit SH, Whetzel CA, Kim K, Femia EE, Almeida DM, Rovine MJ (2014). Daily stressors and adult day service use by family caregivers: Effects on depressive symptoms, positive mood, and dehydroepiandrosterone-sulfate. Am J Geriatr Psychiatry.

[87] Pinquart M, Sörensen S (2005). Ethnic differences in stressors, resources, and psychological outcomes of family caregiving: a meta-analysis. Gerontologist.

[88] Morgan T, Ann Williams L, Trussardi G, Gott M (2016). Gender and family caregiving at the end-of-life in the context of old age: A systematic review. Palliat Med.

[89] Meyer OL, Nguyen KH, Dao TN, Vu P, Arean P, Hinton L (2015). The sociocultural context of caregiving experiences for Vietnamese dementia family caregivers. Asian Am J Psychol.

[90] Polenick CA, Struble LM, Stanislawski B, Turnwald M, Broderick B, Gitlin LN (2018). “The Filter is Kind of Broken”: family caregivers’ attributions about behavioral and psychological symptoms of dementia. Am J Geriatr Psychiatry.

[91] Gallagher-Thompson D, Choryan Bilbrey A, Apesoa-Varano EC, Ghatak R, Kim KK, Cothran F (2020). Conceptual framework to guide intervention research across the trajectory of dementia caregiving. Gerontologist.

[92] Knight BG, Sayegh P (2010). Cultural values and caregiving: the updated sociocultural stress and coping model. J Gerontol Ser B Psychol Sci Soc Sci.

[93] McLean KC, Shucard H, Syed M (2017). Applying the master narrative framework to gender identity development in emerging adulthood. Emerg Adulthood.

[94] McLean KC, Pasupathi M (2012). Processes of identity development: where i am and how i got there. Identity.

[95] Friedemann ML, Buckwalter KC (2014). Family caregiver role and burden related to gender and family relationships. J Fam Nurs.

[96] Kashdan TB, Rottenberg J (2010). Psychological flexibility as a fundamental aspect of health. Clin Psychol Rev.

[97] Shifren K. Caregiving identity and flexibility. In: Identity flexibility during adulthood: perspectives in adult development: Springer International Publishing; 2017. p. 289–301. Available from: /record/2017–45299-018. Cited 2021 Jan 9.

[98] Burdge BJ. Bending gender, ending gender: theoretical foundations for social work practice with the transgender community. Soc Work. 2007;52(3):243–250. Available from: https://pubmed.ncbi.nlm.nih.gov/17850032/. Cited 2021 Feb 24. 10.1093/sw/52.3.243 17850032

[99] Ussher JM, Sandoval M (2008). Gender differences in the construction and experience of cancer care: The consequences of the gendered positioning of carers. Psychol Health.

[100] Cheng HG, Phillips MR (2014). Secondary analysis of existing data: opportunities and implementation. Shanghai Arch Psychiatry.

