# Supplementary material for: Gendered experiences of providing informal care for older people: a systematic review and thematic synthesis
Source: BMC Health Serv Res. 2021 Jul 23;21:730. doi: 10.1186/s12913-021-06736-2 (PMC8306003; doi:10.1186/s12913-021-06736-2)
Supplement: Supplementary file 1 — Additional file 1. [file 12913_2021_6736_MOESM1_ESM.docx]

**Additional Material 1.**

**PRISMA Checklist**

| **Section/topic** | **#** | **Checklist item** | **Reported on page #** |
| --- | --- | --- | --- |
| **TITLE** | | |  |
| Title | 1 | Identify the report as a systematic review, meta-analysis, or both. | 1 |
| **ABSTRACT** | | |  |
| Structured summary | 2 | Provide a structured summary including, as applicable: background; objectives; data sources; study eligibility criteria, participants, and interventions; study appraisal and synthesis methods; results; limitations; conclusions and implications of key findings; systematic review registration number. | 2-3 |
| **INTRODUCTION** | | |  |
| Rationale | 3 | Describe the rationale for the review in the context of what is already known. | 3-4 |
| Objectives | 4 | Provide an explicit statement of questions being addressed with reference to participants, interventions, comparisons, outcomes, and study design (PICOS). | 4-5 |
| **METHODS** | | |  |
| Protocol and registration | 5 | Indicate if a review protocol exists, if and where it can be accessed (e.g., Web address), and, if available, provide registration information including registration number. | 5 |
| Eligibility criteria | 6 | Specify study characteristics (e.g., PICOS, length of follow-up) and report characteristics (e.g., years considered, language, publication status) used as criteria for eligibility, giving rationale. | 7 & Table 3 |
| Information sources | 7 | Describe all information sources (e.g., databases with dates of coverage, contact with study authors to identify additional studies) in the search and date last searched. | 6 &  Table 3 |
| Search | 8 | Present full electronic search strategy for at least one database, including any limits used, such that it could be repeated. | Table 2 |
| Study selection | 9 | State the process for selecting studies (i.e., screening, eligibility, included in systematic review, and, if applicable, included in the meta-analysis). | 6 |
| Data collection process | 10 | Describe method of data extraction from reports (e.g., piloted forms, independently, in duplicate) and any processes for obtaining and confirming data from investigators. | 9-10 |
| Data items | 11 | List and define all variables for which data were sought (e.g., PICOS, funding sources) and any assumptions and simplifications made. | Table 4 |
| Risk of bias in individual studies | 12 | Describe methods used for assessing risk of bias of individual studies (including specification of whether this was done at the study or outcome level), and how this information is to be used in any data synthesis. | 9 &  Table 4 |
| Summary measures | 13 | State the principal summary measures (e.g., risk ratio, difference in means). | 6 &  Table 3 |
| Synthesis of results | 14 | Describe the methods of handling data and combining results of studies, if done, including measures of consistency (e.g., I^2^) for each meta-analysis. | 5 &  Table 1. |

**PRISMA checklist**. *Continued*

| Risk of bias across studies | 15 | Specify any assessment of risk of bias that may affect the cumulative evidence (e.g., publication bias, selective reporting within studies). | 19- 20 &  Table 4 |
| --- | --- | --- | --- |
| Additional analyses | 16 | Describe methods of additional analyses (e.g., sensitivity or subgroup analyses, meta-regression), if done, indicating which were pre-specified. | n/a |
| **RESULTS** | | |  |
| Study selection | 17 | Give numbers of studies screened, assessed for eligibility, and included in the review, with reasons for exclusions at each stage, ideally with a flow diagram. | Figure 1. |
| Study characteristics | 18 | For each study, present characteristics for which data were extracted (e.g., study size, PICOS, follow-up period) and provide the citations. | Table 4 &  Supplementary 2 &  Supplementary 3 |
| Risk of bias within studies | 19 | Present data on risk of bias of each study and, if available, any outcome level assessment (see item 12). | 19- 20 &  Table 4. |
| Results of individual studies | 20 | For all outcomes considered (benefits or harms), present, for each study. | Figure 2. |
| Synthesis of results | 21 | Present results of each meta-analysis done, including confidence intervals and measures of consistency. | 10- 15 |
| Risk of bias across studies | 22 | Present results of any assessment of risk of bias across studies (see Item 15). | 19- 20 &  Table 4 |
| Additional analysis | 23 | Give results of additional analyses, if done (e.g., sensitivity or subgroup analyses, meta-regression [see Item 16]. | n/a |
| **DISCUSSION** | | |  |
| Summary of evidence | 24 | Summarise the main findings including the strength of evidence for each main outcome; consider their relevance to key groups (e.g., healthcare providers, users, and policy makers). | 15-19 |
| Limitations | 25 | Discuss limitations at study and outcome level (e.g., risk of bias), and at review-level (e.g., incomplete retrieval of identified research, reporting bias). | 19-20 |
| Conclusions | 26 | Provide a general interpretation of the results in the context of other evidence, and implications for future research. | 20-21 |
| **FUNDING** | | |  |
| Funding | 27 | Describe sources of funding for the systematic review and other support (e.g., supply of data); role of funders for the systematic review. | 22 |

**PRISMA checklist.** *Continued*

***ENTREQ statement.*** *Enhancing transparency in reporting the synthesis of qualitative research.*

| **No** | | **Item** | | **Guide and description** | **Reported on page** | |
| --- | --- | --- | --- | --- | --- | --- |
| **1** | | Aim | | State the research question the synthesis addresses | p. 4-5 | |
| **2** | | Synthesis methodology | | Identify the synthesis methodology or theoretical framework which underpins the synthesis, and describe the rationale for choice of methodology (e.g., meta-ethnography, thematic synthesis, critical interpretive synthesis, grounded theory synthesis, realist synthesis, meta-aggregation, meta-study, framework synthesis) | p. 6  Table 1. | |
| **3** | | Approach to searching | | Indicate whether the search was pre-planned (*comprehensive search strategies to seek all available studies)* or iterative (*to seek all available concepts until they theoretical saturation is achieved)*. | p. 6  Table 1. | |
| **4** | | Inclusion criteria | | Specify the inclusion/exclusion criteria *(e.g., in terms of population, language, year limits, type of publication, study type).* | p.6  Table 3. | |
| **5** | | Data sources | | Describe the information sources used (e.g., *electronic databases, grey literature databases, relevant organisational websites, experts, information specialists, generic web searches, hand searching, reference lists)* and when the searches conducted; provide the rationale for using the data sources. | p. 6  Table 3. | |
| **6** | | Electronic search strategy | | Describe the literature search (e.g., provide electronic search strategies with population terms, clinical or health topic terms, experiential or social phenomena related terms, filters for qualitative research, and search limits) | Table 2.  Table 3. | |
| **7** | | Study screening methods | | Describe the process of study screening and sifting *(e.g., title, abstract and full text review, number of independent reviewers who screened studies).* | p. 6 | |
| **8** | | Study characteristics | | Present the characteristics of the included studies (e.g., year of publication, country, population, number of participants, data collection, methodology, analysis, research questions). | Table 4.  Supplementary 3. | |
| **9** | | Study selection results | | Identify the number of studies screened and provide reasons for study exclusion (e.g., for comprehensive searching, provide numbers of studies screened and reasons for exclusion indicated in a figure/flowchart). | Figure 1 | |
| **10** |  | | Rationale for appraisal | Describe the rationale and approach used to appraise the included studies or selected findings (e.g., assessment of conduct (validity and robustness), assessment of reporting (transparency), assessment of content and utility of the findings). | | p. 9 |
| **11** |  | | Appraisal items | State the tools, frameworks and criteria used to appraise the studies or selected findings *(e.g. Existing tools: CASP, QARI, COREQ, Mays and Popereviewer developed tools; describe the domains assessed: research team, study design, data analysis and interpretations, reporting).* | | p. 9 |
| **12** |  | | Appraisal process | Indicate whether the appraisal was conducted independently by more than one reviewer and if consensus was required. | | p. 9 |
| **13** |  | | Appraisal results | Present results of the quality assessment and indicate which articles, if any, were weighted/excluded based on the assessment and give the rationale. | | p. 9  Table 4. |
| **14** |  | | Data extraction | Indicate which sections of the primary studies were analysed and how were the data extracted from the primary studies? (e.g., all text under the headings “results /conclusions” were extracted electronically and entered into a computer software). | | p. 9-10 |
| **15** |  | | Software | State the computer software used, if any | | p. 9 -10 |
| **16** |  | | Number of reviewers | Identify who was involved in coding and analysis. | | p. 9 -10 |
| **17** |  | | Coding | Describe the process for coding of data (e.g., line by line coding to search for concepts). | | p. 9- 10 |
| **18** |  | | Study comparison | Describe how were comparisons made within and across studies (e.g., subsequent studies were coded into pre-existing concepts, and new concepts were created when deemed necessary). | | p. 9 -10 |
| **19** |  | | Derivation of themes | Explain whether the process of deriving the themes or constructs was inductive or deductive | | p. 9 -10 |
| **20** |  | | Quotations | Provide quotations from the primary studies to illustrate themes/constructs, and identify whether the quotations were participant quotations of the author’s interpretation | | Supplementary 2. |
| **21** |  | | Synthesis output | Present rich, compelling, and useful results that go beyond a summary of the primary studies (e.g., *new interpretation, models of evidence, conceptual models, analytical framework, development of a new theory or construct).* | | p. 10-15 |

***ENTREQ statement.*** *Continued*
