# Supplementary material for: Gendered experiences of providing informal care for older people: a systematic review and thematic synthesis
Source: BMC Health Serv Res. 2021 Jul 23;21:730. doi: 10.1186/s12913-021-06736-2 (PMC8306003; doi:10.1186/s12913-021-06736-2)
Supplement: Supplementary file 2 — Additional file 2. [file 12913_2021_6736_MOESM2_ESM.docx]

**Additional Material 2.** *Data extraction of the studies' authors' key findings and interpretations and correspondent participants' quotations.*

| **Study**  **Authors' findings** | **How personal control mediates suffering: Elderly husbands' narratives of caregiving.**  ***Black et al., 2008.*** |
| --- | --- |
| ***From abstract:*** Three thematic "tools" of control emerged as strategies men used to mediate their suffering: 1) the power of the little; 2) preserving self-identity and marriage-identity and, 3) finding purpose in the role of caregiver. | |
| ***Finding 1 The power of the little.***  ***Participants' quotations:*** "Give me a foot, put it up on me, I'll take your shoe off. But if you get my knee dirty, you're going to wash my leg." She says, "It would be an honor. And she really meant it, the way she said it".  ***Authors' interpretation:*** Theme 1 shows how the "little" of everyday conversations, incidents, or tasks have the power to give hope, change lives, or predict death. A moment of clarity in the wife, dressing her with ease, understanding nuance in her voice, and keeping a link with the "outside," gave elderly caregiving men a sense of control over their lives. Conversely, when the "little" of a wife's comprehension or ability to maneuver through the home lessened, the potential for husbands to maintain control decreased***.*** | |
| ***Finding 2 Preserving self-identity and marriage-identity.***  ***Participants' quotations:*** "If I don't keep moving and shaking, ennui will set in. Grief will set in"  ***Authors' interpretation:*** Theme 2 shows that husbands used long-standing qualities, such as faith, perseverance, and intelligence, and new attributes, such as acceptance, compassion, and "living one day at a time" to manage the emotional difficulties of caregiving. Men also used masculinities, or behaviors they viewed as gender appropriate, such as preparedness, knowledge, and silence, to defend decisions they made as caregivers. Men brought "appropriate" long-standing and recent characteristics together to maintain and foster existing self- and marriage-identities. | |
| ***Finding 3 Finding purpose in the role of caregiver.***  ***Participants' quotations****:* "What am I going to do?" One step at a time. One day at a time. I am here for a certain reason, I guess. Maybe I am here for Marie. Marie's here for me." "The moral of my story is: He tried, he tried."  ***Authors' interpretation:*** Theme 3 shows that caregiving did not impede existential tasks but provided answers to men's queries about purpose in life. In the latter stage of life, caregiving became not only a symbol of each man's commitment to his wife, but also a reason why each man had lived as long as he had. Their lives' major purpose was clear—to remain their wife's caregiver until death. | |

| **Additional Material 2.** *Continued* | | |
| --- | --- | --- |
| **Study**  **Authors' findings** | **Elderly husbands caring at home for wives diagnosed with Alzheimer's disease: are male caregivers different?**  ***Cahill, 2000*** | |
| ***From abstract:*** Results show how men demonstrated a strong injunction to care, performed intimate personal care tasks competently, received limited government support and derived some satisfaction from the caregiving role. Despite similarities between male and female caregivers being noted, some gender differences in the way in which men approached the care role are described. | | |
| ***Finding 1 Reasons for caring.***  ***Participants' quotations:*** "She's my wife and partner, we've done everything together for 41 years, you take your vows. ***Authors' interpretation:*** Main reasons for caring included love, marriage, duty or a combination of each. For many caring was also seen as a relational activity. | | |
| ***Finding 2 Caring tasks performed.***  ***Participants' quotations:*** -. ***Authors' interpretation:*** For the majority, it seems the care role was modelled on earlier work roles. | | |
| ***Finding 3 Incontinence management.***  ***Participants' quotations:*** "It's part of what has to be done, you just get on with it”. ***Authors' interpretation:*** When asked how they coped, the majority (71 %) reported they considered this as a routine aspect of care and approached it pragmatically as a job which had to be done. | | |
| ***Finding 4 Formal support service and caregiving.***  ***Participants' quotations:*** -. ***Authors' interpretation:*** In general, male caregivers received limited formal support with the physical and personal care services. | | |
| ***Finding 5 Informal support services and caregiving.***  ***Participants' quotations:*** "My daughter helps, she gives me quite a lot because she's a barmaid and she works odd hours". ***Authors' interpretation:*** Some were defensive about their children's involvement in parent care. | | |
| ***Finding 6 Satisfaction with the care role.***  ***Participants' quotations:*** "I mean I'm trying to give her better than she'd have if she were to go to a home. I am keeping her alive, that is what we both agreed on. I feel if I can share with her as much as I can she will have a longer fun.". "It's satisfying because I still! have her with me, that's the name of the game' or from having their wives occasionally express gratitude to them”. ***Authors' interpretation:*** Interestingly, a major source of satisfaction was seeing their loved one remaining healthy and looking happy. Caregivers seemed to attribute their spouses' health and wellbeing to the individually designed care they could provide at home. One quarter of the sample acknowledged that for them a source of satisfaction was the knowledge that they were responsible for either keeping their marriage and relationship together. | | |
| **Study**  **Authors' findings** | **Spousal caregiving and crossing gender boundaries: Maintaining gendered identities**  ***Calasanti and Bowen, 2006*** | |
| ***From abstract:*** In exploring the ways gender might influence caregiving, we find that husbands and wives both cross gender boundaries in order to give care to their spouses; and such traversing presents different challenges to each. | | |
| ***Finding 1 Taking over new household tasks***  ***Participants' quotations:*** Caregiving wives:" Of course I never had to put gas in a car, I never had to check the tires, I never had to do anything about an automobile because he did everything. And now I have that and that is hard."  Caregiving husbands:" I found out how difficult it is to do all these things”.  ***Authors' interpretation:*** In this study, gender boundaries were often crossed in relation to the usual division of household labor***.*** In both cases, whether male or female, spouses have to take over new tasks to allow the couple to survive as a unit. Overall, it appears that most husbands faced greater challenges than caregiving wives in having to adopt more daily tasks, and they derived a variety of techniques for managing these. They learned to perform some, if not all, of these tasks themselves; received help from other family members; or they paid for others to manage some of these tasks. | | |
| ***Finding 2 Tasks performed to maintain gender identity: Gendered appearance and gendered sense of self.***  ***Participants' quotations:*** Caregiving wives: «He always loved to look good, you know, and took care of his body and stuff…And I feel like I need for him to look as good. If he doesn’t want to shave, I send him back [to do it]." "I have got to give him a little money, you know, to carry. I am not going to tell him he can't do that because that would really hurt his spirits….". Caregiving husbands:" I use this soap, you know, that is in a bottle that you buy, it's a good grade. It's ladies' soap, and just squeeze a few drops in there and that is what I give her a bath with that … And then dry her off good, and I put a good grade of lotion on her." "I said yeah you made that really look good you know and agree with her to make her feel good. She will grin. Just let her take credit. It doesn’t hurt a thing."  ***Authors' interpretation:*** While both husbands and wives help their spouses maintain gendered appearances, differences also emerged. For wives, this behavior entailed an extension of activities they were used to performing: a variety of personal care tasks for others, such as bathing and grooming and making sure others are properly dressed in clean and matching clothing. For men, however, these are generally new tasks: forms of bodily care for another that they were not used to. | | |
| **Study**  **Authors' findings** | **Taking 'Women's Work’ 'Like a Man': Husbands' Experiences of Care Work**  ***Calasanti and King, 2007*** | |
| ***From abstract:*** We found that these husbands' approaches to caregiving and their strategies for dealing with the work and feelings involved were rooted in their sense of selves as men. | | |
| ***Finding 1 Husbands' Approaches to Care: "Pick it up Like You Do a Trade.***  ***Participants' quotations:*** "At ﬁrst ... when you start taking care of a woman, you know, you don't know exactly how to do it". "And] the ladies at the day care center ... say, "[Jerry] you do an excellent job."  ***Authors' interpretation:*** Men's relative lack of stress resulted not from a refusal on their part to perform difﬁcult work but rather from their relative freedom from responsibility for particular expectations or from their wives' feelings. | | |
| ***Finding 2 Dealing with Care Receivers: "If She Needed a Shower, I Gave Her a Shower".***  ***Participants' quotations:*** "She is so determined that she is really powerful ... and I have got a lot of scars on me because of that.. . And I ... try my best to keep her in a good mood. [But] I can tell when she is not going to give up and then ... I will say okay, [and] start taking [her clothes] off. And so, I have to forcefully ..."  ***Authors' interpretation:*** Thus, in reporting ways in which masculine identities might have led men to attempt enforcement strategies that caregiving wives avoided, we are not making a case for better or worse care. Instead, our focus is only on how strategies ﬂow from gender repertoires, and the importance of understanding the structural bases for these repertoires when designing interventions. | | |
| ***Finding 3 Dealing with Caregivers' Stress/Feelings.***  ***Participants' quotations:*** "I just keep my cool and go on. She uses a word that starts with a h and a d and a c. I just keep cool. ... I just keep it cool and do not say anything back to her and go on, and just sit tight. ..."‘’ I try to get back to some form of being ‘‘normal’’: hunting, ﬁshing.” “You can go read your e-mail and do some research ... I subscribe to a great magazine and it’s fun to read about the archaeology review ... And so, reading is a diversion.” “Most men keep things to themselves. I am not going to tell everybody I know that I am stressing, or I feel depressed.” “I just go get another beer.”  ***Authors’ interpretation:*** These strategies—exerting force, focusing on tasks, blocking emotions, minimising disruption, distracting attention, and self-medicating—ﬁt the structural positions of working-, middle-, and professional-class men, whose occupational roles taught them the values of independence and pride in skills. | | |
| **Study**  **Authors ‘findings** | ***The Impact of Caregiving: Older Women’s Experiences of Sexuality and Intimacy***  ***Drummond et al. 2013*** | |
| ***From abstract:*** Findings suggest that older women’s sexual identities and experiences of sexuality are marked by both ageist constructions of ‘‘older woman as asexual’’ and loss of self-associated with taking on the role of caregiver. Findings also suggest that women resist these constructions of self through the development of other forms of intimacy in their lives. | | |
| ***Finding 1 Shifting Identities***  ***Participants’ quotations:*** “Him being sick and me being a caregiver have taken over. I know it’s not supposed to for me, I try to do other things,  but that’s sort of the overwhelming part of our identities. So, it’s hard to work ourselves out of it and go back to some sort ofyou know who we were.” “‘‘how could I possibly imagine sleeping with him anymore when I’m wiping his behind’’. It’s not pleasant.”  ***Authors’ interpretation:*** In summary, most spouses in this study could describe a moment in time when they became aware of a shift in their multiple identities directly connected to the shift in their role from spouse to that of caregiver*.* | | |
| ***Finding 2 Women’s Agency Expressed Through Intimacy***  ***Participants’ quotations:*** “We try to, you know, we try to kiss and hug but R. sits here and I have to bend down and he’s always liked breasts so we try to sort of do something but um it’s not really, it’s playful. I mean it’s not what you would call overtly sexual. It’s nice.”  ***Authors’ interpretation:*** In summary, caregivers adapted to the changes resulting from their own and their spouses’ shifts in identity by developing new ways of expressing intimacy in order to create situations in which some of their needs could continue to be met. | | |
| ***Finding 3 Talking to Others About Sexuality and Intimacy***  ***Participants’ quotations:*** “He [my doctor] says things like ‘down there’ referring to vaginas, my family doctor. He is sort of my age and it’s awkward for him I guess ... given that you can see that he doesn’t ask too much about our sex lives.”  ***Authors’ interpretation:*** In summary, considering participant’s narratives of sexuality as ‘‘irrelevant’’ and service provider’s assumptions and discomforts, it is not surprising that sexuality is a topic absent in the caregiver and health care service provider encounter. | | |
| **Study**  **Authors ‘findings** | ***Experiences of long-term home care as an informal caregiver to a spouse: gendered meanings in everyday life for female carers***  ***Eriksson et al., 2013*** | |
| ***From abstract:*** The ﬁndings of this study show that the informants frequently reﬂected on their caregiving activities in terms of both general and heteronormative expectations. | | |
| ***Finding 1 Hetero-polarisation in the relationship***  ***Participants’ quotations:*** “There is no point in arguing, to say anything. In this case, for the woman it is ordered silence in the assembly, a sense of reason doesn’t exist. I’ll think it is hard, it really is.”  ***Authors’ interpretation:*** The women felt there were no ‘rational’ arguments they could present to their husbands, family or friends for meeting their own needs; rather, their partners’ cognitive impairment took precedence. | | |
| ***Finding 2 Introspection connected to caring activity***  ***Participants’ quotations:*** “It is typical for women, it’s in our genes to take care of everything and constantly worrying about everything, it is like all the world’s problems are resting on your shoulders. But for men, they seem to go along no matter what, because they got the women to rely on.” “And my girls, they are coming and going like frequent ﬂyers in our house, one of them lives a two-hour drive away and the other lives about three hours away, still they’re doing everything they can to support, help and arrange our lives to the best, but it isn’t easy.”  ***Authors’ interpretation:*** Many different arrangements can be made to keep the personal responsibility for the caring within the ‘family’. The nurturing skills associated with each of the different roles throughout a woman’s lifetime, such as ‘daughter’, ‘wife’ and ‘mother’, provide support, maintain continuity, and can be regarded as important resources for both the family and society in general. | | |
| **Study**  **Authors ‘findings** | ***Beyond Familism: A Case Study of the Ethics of Care of a Latina Caregiver of an Elderly Parent with Dementia***  ***Flores et al., 2009*** | |
| ***From discussion:*** Ana’s narrative shows the struggle of a second-generation, working-class Latina daughter to conform to a Latino ethics of care, to negotiate the cultural mandates of familism, and to provide compassionate care for her mother. | | |
| ***Finding 1 Understanding Her Mother’s Illness***  ***Participants’ quotations:*** “She has always been a stressful person.”  ***Authors’ interpretation:*** Ana’s explanatory model of her mother’s illness is consistent with other accounts of how Latino caregivers and family members “explain” the cognitive and behavioral symptoms of dementia as being related to “nerves” or long-standing personality traits. Ana’s nativity in the United States, her awareness of dementing illnesses, and her belief that her mother suffers from dementia, she relies on a “cultural” explanation for her mother’s behaviors. | | |
| ***Finding 2 On Becoming a Caregiver***  ***Participants’ quotations:”*** My brothers are kind of in their own little world, you know. I think it has to do with the culture a lot of it, you know, how they are brought up. Like my Mom has always had this thing that men are to be catered to and women are pretty basically on their own.”  ***Authors’ interpretation:*** While female caregiving of elders is not a uniquely Latino phenomenon, Ana assumes the primary caregiver role because she sees it as a cultural mandate rooted in familistic values; thus, she carries forward the tradition by assuming the caregiver role for her mother. | | |
| ***Finding 3 Model of Care***  ***Participants’ quotations:*** “When she gets verbally (abusive), sometimes I will listen, but then there are other times that I just, I ﬁnd myself losing it to be honest, you know. I just answer back, you know, and then she becomes more ... and I try, you know, to be more on top of it. I just walk away. I just walk away, and she will continue.”  ***Authors’ interpretation:*** Ana reported relying on her love for her mother to remain patient and compassionate. She alternates between accepting her caregiver role as an obligation, given her cultural upbringing, and wanting to run away and escape her mother’s outbursts, which she sometimes does by locking herself in her room. | | |
| **Study**  **Authors ‘findings** | ***Releasing from the Oppression: Caregiving for the Elderly Parents of Japanese Working Women***  ***Hashizume, 2010*** | |
| ***From abstract:*** I generated a substantive grounded theory, resulting in the identification of the core concept of “releasing self,” which included three dimensions: laughing away, self-belief, and losing enthusiasm for the elderly and elderly care. | | |
| ***Finding 1 Following the Norm Automatically***  ***Participants’ quotations:*** “When I started taking care of my mother-in-law, I was the only one to handle it. Both my husband and my daughter had jobs.”  ***Authors’ interpretation:*** They performed all of their tasks without complaining. | | |
| ***Finding 2 Reducing the Amount of Tasks***  ***Participants’ quotations:*** “Ten years have passed since I started taking care of my mother. I am also getting older and cannot give her [a] bath alone.”  ***Authors’ interpretation:*** All the caregivers discovered their limits of power and stamina to manage their numerous tasks alone. | | |
| ***Finding 3 Attributing the Choice of New Coping Strategies to Change with the Times***  ***Participants’ quotations:*** “Some elderly people dislike seeing men in the kitchen, but nowadays men and women are equal.”  ***Authors’ interpretation:*** Caregivers recognised that meeting the needs of their husband and the elderly parent was incompatible with the continuance of the woman’s own work and the valuing of her personal life. | | |
| ***Finding 4 Feeling Oppressed***  ***Participants’ quotations:*** “Because I am so busy with work and caring for my mother, my home is an untidy mess. That makes me feel ashamed.”  ***Authors’ interpretation:*** This step, feeling oppressed, involved five categories. Three of them, “feeling sorry,” “feeling guilty,” and “feeling ashamed” represent a caregiver’s negative feelings about herself. Those feelings arose from remorse for having gone against the norm. The other two categories, “feeling angry” and “feeling disgusted,” represent the attitudes of the caregivers directed against the caregiver’s unreasonable conditions. | | |
| ***Finding 5 Releasing self***  ***Participants’ quotations:*** “Even though I implore my husband about gender equality, he never puts his hands to housework. I sometime tease him and say, “How would you survive by yourself if I get sick and go into hospital?”  ***Authors’ interpretation:*** Releasing self includes three dimensions: laughing away, self-belief, and losing enthusiasm for the elderly and elderly care. | | |
| ***Finding 6 Making Accomplishments***  ***Participants’ quotations:”*** When I get paid, I feel I am evaluated exactly and that gives me energy for tomorrow.”  ***Authors’ interpretation:***  Successfully managing these demands brought the caregivers a substantial sense of achievement and psychosocial reward. | | |
| **Study**  **Authors’ findings** | **Living and loving with dementia: Negotiating spousal and caregiver identity through narrative**  **Hayes et al., 2009** | |
| ***From abstract:*** The intensive interviews conﬁrmed that identity change on the part of sick spouses had important implications for intimacy, although not always in adverse ways. | | |
| ***Finding 1 How ADRDs affect intimacy.***  ***Participants’ quotations:*** Caregiver husband: ““Some of your friends just kind of hold you at arm's length when you've got, your spouse has Alzheimer's disease.” Caregiver wife: “And it's a little embarrassing to be someplace and people are looking at him like.”  **Authors’ interpretation:** The meaning of sexual intimacy and its relation to self- identity was at least partially structured by gender. While husbands frequently struggled with their continued desire to have intercourse with an increasingly impaired spouse, wives often resented their continued sexual intimacy with husbands who were no longer seen as husbands, but children, or empty bodies or different, less desirable persons***.*** | | |
| ***Finding 2 The role of gender in structuring intimacy***  ***Participants’ quotations:*** Caregiver husband: “She's lost memory skills, she's lost some certain physical skills, she can't focus in on things, but Kay's still Kay.” Caregiver wife: “I’m tired. I do not want sex. ‘I don't want sex with you because you're not who you were before.”  **Authors’ interpretation**: Men expressed changes in their level of sexual intimacy due to the effects of the ADRD on their wives, the breaking down of bodily functions and changes in appearance were the principle reasons they deﬁned their wives as “less sexy,” and not being a “woman ﬁrst.” Husbands often expressed frustration over discontinued sexual intimacy while wives expressed frustration over a lack of emotional, and intellectual and instrumental reciprocity as sexual intimacy continued***.*** | | |
| ***Finding 3 The importance of reciprocity in retaining intimacy***  ***Participants’ quotations:*** Caregiver husband:” I couldn't get her interested enough to where I felt comfortable proceeding. . . that's one of the functions that's just left her. I mean to know actually what it [sexual intimacy] even was.” Caregiver wife:” ‘Okay you can't do anything during the day, and you say you can't ﬁgure out anything, but you still want sex.’ I mean ...well like he says, ‘I can't, you know, I can't balance a checkbook. I cannot do this, and I can't remember things. I can't ﬁx my own meal,’ but still you want intimacy. You want sex. In that area your memory is just ﬁne.”  ***Authors’ interpretation:*** *For the caregiver wives who did continue to share sexual intimacy with their husbands, they often resented the lack of emotional reci*procity and their husband's inability to help in daily household activities while they were expected to continue to fulﬁll their “wifely duties.” The most important aspect of self-disclosure for men was that it served to indicate spousal consent, while for women it represented emotional, intellectual, and pragmatic involvement in activities outside of sexual intercourse. | | |
| **Study**  **Authors ‘findings** | ***Responding to Symptoms of Alzheimer’s Disease: Husbands, Wives, and the Gendered Dynamics of Recognition and Disclosure***  ***Hayes et al., 2010*** | |
| ***From abstract:*** Men were slower to recognise the symptoms of ADRDs, with social others usually bringing the problems to their attention. They often attributed symptoms to a less-problematic cause and engaged in extended normalisation of their wife’s condition. Women were quicker to recognise symptoms and often noticed subtle changes in their husbands but failed to take action quickly. They were reluctant to disclose their concerns to their impaired husbands, which might have protected the husband’s masculine identity and served to maintain the wife’s own sense of self in relation to him. We suggest that husbands were able to normalise because the wife’s symptoms did not change marital authority dynamics, but authority relations were reversed by the illness for caregiver wives. | |  |
| ***Finding 1 Identifying Moments***  ***Participants’ quotations:*** Caregiver husband:’’ She just would not quit working.” Caregiver wife: ’The first thing that happened [was that] he had always had the ability to put the eight people in the square back together again. It was always appreciated that he was able to do that. I noticed that he was not doing that anymore and I thought, how strange.”  ***Authors’ interpretation***: In general, caregiver husbands were likely to engage in extended normalisation and appeared initially to incorporate their wife’s symptoms into the customary flow of marital interaction. The dynamics of symptom recognition and response for wives tended to differ sharply as they quickly recognised the behavioral changes but were slow to act on them. Caregiver wives were quicker to interpret behavioral changes as cognitively related but expressed a reluctance to confront their husbands about their interpretations. | |  |
| ***Finding 2 Acting on symptoms***  ***Participants’ quotations:*** Caregiver wife: “He went through a period of time in which he was very emotional. He would cry and he would say, “What is wrong with me?” And I just kind of told a therapeutic lie. I said, “Do you remember when you had that bleeding in your head?” And he could relate to that. And I said, “That did damage to your nerves and brain cells in your brain. And some of that is nothing that you can help, nothing that can be corrected… It’s just there… And we’ll work together as a team and get through it.”  ***Authors’ interpretation:*** Caregiver wives’ decisions to withhold the diagnosis from cognitively impaired husband might have served to protect the status and identity of husbands and, in turn, women’s own identities as wives. Consistent with prior research, men were generally saddened but accepting of the changes they observed, and proud that they were able to care for their wife. | |  |
| **Study**  **Authors ‘findings** | ***Discourse-derived perspectives: Differentiating among spouses’ experiences of caregiving***  ***Hepburn et al., 2002*** | |
| ***Authors’ findings***  ***From abstract:*** Results partly confirmed previous findings that wife caregivers are more distressed than husbands, but the results also indicated these caregivers were more similar than dissimilar. | | |
| ***Finding 1 Occurrence of coded expressions (gender focus)***  ***Participants’ quotations:*** no gender assigned: “The next time he does something, I’m going to get mad and finally I’m going to blow up.” “.”  ***Authors’ interpretation:*** We were surprised to note the low frequency with which caregivers mentioned certain themes noted in the literature on caregiving. For example, there was almost no mention of the rewards of caregiving, a very low frequency of mention of spiritual dimensions for their activities, and infrequent mentions of the use of humor or the need for additional knowledge for caregiving. | | |
| ***Finding 2 Searching for differentiating factors (gender focus)***  ***Participants’ quotations:*** Caregiver wife:” it’s like a constant death.”  ***Authors’ interpretation:*** Other results support the gender-based view of caregiving impact but admit to alternative explanations. | | |
| ***Finding 3 Comparisons on other dimensions***  ***Participants’ quotations:*** *Caregiver husbands:” I don’t mind helping her and doing things for her and that. Um, I think that if you love somebody you just take those things as something that you do or that you are willing to do, you know. So, that’s what I do.”*  *Caregiver wives:” Um, it’s been real frustrating.”*  ***Authors’ interpretation****:* We labeled the framing categories relational (linked with “couple identity affirmation”), instrumental (“more work”), role acquiring (“acquisition of new role”), and reactive (where the transcript contained none of the three codes, but spouses described their caregiving experience in terms of their reaction to the losses associated with dementia). Relational spouses seemed in much better condition than those in the other groupings, indicating a balanced and engaged life with their spouse. Reactive spouses appear more ambivalent than either the relational or the instrumental spouses. They continued to express a felt connection with their spouse, but also a sense of loss of relationship with the spouse. Role acquiring spouses were the saddest of all the groups, but also expressed a sense of satisfaction in their own development. Like the reactive spouses, the role acquiring spouses spoke more frequently about losing the spouse they knew, but they also mentioned continued shared activities with the spouse. | | |
| **Study**  **Authors ‘findings** | ***Developing a Cultural Model of Caregiving Obligations for Elderly Chinese Wives***  ***Holroyd, 2005*** | |
| ***From abstract:*** The model proposed for interpreting elderly Chinese wives’ caregiving obligations highlights the tension-filled links between Confucianism and government guidelines, early and ongoing socializing experiences, and self-identity. | | |
| ***Finding 1 Marital Duty-******Bound Roles and Responsibilities***  ***Participants’ quotations:*** “It’s my role as a wife.”  ***Authors’ interpretation:*** Behaviors such as caregiving may be so deeply internalized, while at the same time may provide a reward and directed role. | | |
| ***Finding 2 Reciprocity and Burden***  ***Participants’ quotations:*** “I have for him all this time and he never really did much for me only some money over the years.”  ***Authors’ interpretation:*** Some wives who were caregivers felt that, within the confines of marriage, they had given enough. | | |
| ***Finding 3 Public Guidelines and Upholding Reputations as Chinese Wives***  ***Participants’ quotations:*** “We Chinese women are very traditional; you stay with your husband until he dies.”  ***Authors’ interpretation:*** The self-identity of a wife who is a caregiver becomes equated with her social position. | | |
| ***Finding 4 Monetary Restrictions***  ***Participants’ quotations:*** “Our family budget is very tight because I had to give up my job.”  ***Authors’ interpretation:*** This lack of finances combined with the lack of public support for their plight means wives who are caregivers are often left with feelings of confusion and hopelessness. | | |
| ***Finding 5 Affection as an Emotional Force to Sustain Caregiving***  ***Participants’ quotations:*** -  ***Authors’ interpretation:*** This couple fully expected a time when the care would reverse again. | | |
| ***Finding 6 Effects of the Caregiving Role***  ***Participants’ quotations:*** “I have to cook for him every day and the food is so tasteless. I tell him to sit on the chair, and he yells at me.”  ***Authors’ interpretation:*** *W*ives emphasized the emotional burden and anxiety associated with the daily grind of caregiving. | | |
| ***Finding 7 The Creation of Self-Identity Through Caregiving***  ***Participants’ quotations:*** “..it has given me a new reason in my old age.”  ***Authors’ interpretation:*** Creation of self-identity through caregiving. | | |
| **Study**  **Authors ‘findings** | **Caregiving Between Two Cultures: An Integrative Experience**  **Jones et al., 2002** | |
| ***From abstract:*** Analysis of interview data led to development of a substantive theory of caregiving between two cultures, reflecting the paradox of living and caregiving by two sets of standards and worldviews. The primary strategies used to manage the caregiving challenges were connecting and calibrating. Through personal growth and finding meaning, the caregivers integrated the caregiver role into their lives and became more connected with their families and within themselves. | | |
| ***Finding 1 Causal Conditions Transplanted Filial Values***  ***Participants’ quotations:*** “It is my duty to take care of them. As Chinese, we received this kind of education.”  ***Authors’ interpretation:*** Their sense of obligation, filial responsibility, and commitment was evident in comments from many participants. | | |
| ***Finding 2 Contextual Conditions in Transition***  ***Participants’ quotations:*** “I am a mother, grandmother, and also a daughter and, of course, a wife. I find it very difficult.”  ***Authors’ interpretation******:*** Cultural assumptions associated with the roles of mother, daughter, and wife were often inherently conflicting between the traditional and new social systems. | | |
| ***Finding 3 Strategies Connecting and Calibrating***  ***Participants’ quotations:*** “A woman who has problems with her husband, I don’t think can go through this . . . Without his support, without his understanding, I could not do it.” “This is planned by someone who can see many things we cannot see. That is God, and He prepared me for this.”  ***Authors’ interpretation:*** Connecting with family resources. This was a common strategy the caregivers used. Help from husbands directly in providing the care and indirectly in handling other tasks that freed the women for caregiving was frequently mentioned. Caregivers acknowledged the support of husbands in welcoming their parents to live with them and in helping financially. The principle of reciprocity in the sharing of filial responsibility was also evident. | | |
| ***Finding 4 Consequences Integration***  ***Participants’ quotations:*** “If everything is smooth along the way, [if] there are no difficulties in your life, you are not going to be strong because you have not experienced battles and have not get the training. Because you have gone through many difficult things, you become a strong person.”  ***Authors’ interpretation:*** In spite of the difficulties associated with caregiving, caregivers found ways to manage the challenge and to integrate the caregiving role into their lives. Some even reported positive outcomes. Many of the caregivers perceived the challenge of caregiving as a stimulus for personal growth. | | |
| **Study**  **Authors ‘findings** | **Older husbands as carers: Constructions of masculinity in context of caregiving**  **Kluczyńska, 2015** | |
| ***From abstract:*** The findings revealed four ways in which older caregivers talk about masculinity, and for all of them hegemonic masculinity was a point of reference. Masculinity was defined not only in relation to the carer’s role, but also old age and the state of men’s health. The ways of perceiving the activity of caring were crucial. Analysis allowed the main motives of providing care to emerge obligation, love, and attachment. The research findings showed that an important factor in the way masculinity is constructed by older men caring for their wives was the definition of care. Men who perceive care as a masculine task feel less frustrated in the care-giver role, and sometimes gain satisfaction and a source of self-esteem from caregiving. | | |
| ***Finding 1 Older men’s motives for caring for their wives***  ***Participants’ quotations:*** “We spent 50 years together… it’s my duty, full stop!” “That’s my wife, I promised till death us do part” “My conscience wouldn’t allow me to act differently, as long as I can still do it”  ***Authors’ interpretation:*** The most common motivation was marital duty. | | |
| ***Finding 2 Household duties and work in the context of caring and the construction of masculinity***  ***Participants’ quotations:*** “I have never distinguished female tasks from male tasks. Work is work, and it’s got to be done.”  ***Authors’ interpretation:*** My analysis showed that men more often indicated the practical dimension of care, whereas the emotional one receded into the background. The practical dimension of care, being more visible and often more time-consuming, might be defined as the more important one. | | |
| ***Finding 3 Construction of care and the carer’s role***  ***Participants’ quotations:*** “Who should do the job of caring? I’m supposed to get another person in to do it?” “We are together, what can I do?”  ***Authors’ interpretation:*** Men have a tendency to describe themselves as the person who cares by way of continuous commitment and responsibility, which is manifested through providing financial support. | | |
| ***Finding 4 Construction of masculinity and care***  ***Participants’ quotations:*** “being a man” “  ***Authors’ interpretation:*** Analysis revealed four ways of talking about masculinity, and for all of these, hegemonic masculinity was a point of reference.” | | |
| **Study**  **Authors ‘findings** | **Self-Characterizations of Adult Female Informal Caregivers: Gender Identity and the Bearing of Burden**  ***Kramer, 2005*** | |
| ***From abstract:*** Results were tabulated and critically examined in relation to stereotypical gender traits, as well as social and political processes that create gender dichotomies. Overall, self-characterizations indicated caregivers had internalized stereotypical female gender traits that support and facilitate the enduring of burden. | | |
| ***Finding 1 Characterizations and female gender identity***  ***Participants’ quotations:*** “Unsure and distrustful, obligated to care, alone, broken, and invisible, resigned and accepting, wise and resourceful, organizer, planner, mother and healer, honest, fair, hopeful with faith, ambivalent, guilty, dependent ,weak, grateful fortunate, privileged, happy, satisfied confident and capable, out of control, needed, extra miler, intuitive, equal, self-caring, not a victim and intact.”  ***Authors’ interpretation:*** Self-characterizations can be interpreted in multiple ways and characterizations that, on the surface, seem inconsistent with female gender identity and can be read as supporting multiple females identified traits | | |
| ***Finding 2 Female gender identity and burden***  ***Participants’ quotations:*** -  ***Authors’ interpretation:*** Women, drawing from self-characterizations provided, have internalized characteristics consistent with a selflessness that is fundamental to the provision of unpaid caregiving labor. Four overarching characteristics of selflessness predominate. These caregivers, on the whole, self-characterized as being (a) open and available to lovingly care, (b) indecisive and uncertain of themselves, (c) compliant and accommodating to the situation, and (d) dutiful and obligated to endure through the course of care provision. | | |
| **Study**  **Authors ‘findings** | ***Concepts of Burden in Giving Care to Older Relatives: A Study of Female Caregivers in a Mexico City Neighborhood***  ***Mendez et al., 2008*** | |
| ***From abstract:*** Burden was a multi-dimensional construct that referred to specific situations that made caregivers feel emotionally or physical “heavy.” Burden also referred to “being a burden” by being in the way, making things difficult, or being a ‘weight’ on caregivers’ shoulders. However, women in this study also viewed burden as a positive sacrifice that involved love, initiative, and good will. This study is an important first step in defining the ways in which caregiving is positively and negatively meaningful for Mexican caregivers and their families. | | |
| ***Finding 1 Physically and emotionally pesado (heavy) situations***  ***Participants’ quotations:*** “tires me a lot. My back gets tired, it makes me pesado.” “Ay! I cannot [do it anymore]. I think my entire body gets tired and sometimes and I [can’t] give but at the same time, I try to revive myself and keep going.” ““Okay, yes, I used to see that it was making me pesado but I didn’t know it. Now, I see it that way, right? It used to be very pesado to take care of her [mother-in-law]. After a while I was getting frustrated because she didn’t want to eat and I almost had to force her to take food, I had to get her get up to go to the bathroom and all that. And you know, I have seen it with my dad [too]. Yes, I feel that, yes, it’s pesado to take care of a sick person.”  ***Authors’ interpretation:*** For the women in this study, burden referred to tangible situations that were emotionally or physically pesado for them. | | |
| ***Finding 2 Ser carga (being a burden)***  ***Participants’ quotations:*** “Let us imagine that I’m 70 years old and she [daughter] is twenty-something and married with children. I’d tell her, ‘listen, daughter, attend to your children, your husband, your life. Don’t worry about me. If I can serve you in some way, I’m here but I’m not going to be a burden on you.’”  ***Authors’ interpretation:*** The women also viewed burden as an undesirable state of dependency (ser carga) that inflicted stress or pain on others. | | |
| ***Finding 3 Burden as sacrifice***  ***Participants’ quotations:*** “this love to do it, this good will that [makes me] want to do it.” “that having to deal with sick persons, for me, is a form of purification, of unification; I don’t see it as a punishment, and I don’t complain. No, for me it has been something positive.”  ***Authors’ interpretation:*** Burden meant sacrificing for others in ways that were rewarding for caregivers. We found that the perceptions of burden differed between younger and older generation caregivers; younger caregivers tended to view burden positively and negatively whereas older caregivers were neither positive nor negative in their assessments of caregiving. | | |
| **Study**  **Authors ‘findings** | ***The Importance of Reciprocity for Female Caregivers in a Super-Aged Society: A Qualitative Journalistic Approach***  ***Paillard-Borg and Stromberg, 2014*** | |
| ***From abstract:*** Reciprocity was identiﬁed as the glue holding the joy and burden of the role of caregiving for elderly parents. Moreover, gender was identiﬁed as a motivator for reciprocity from a macro to a micro level in a super-aged society. | | |
| ***Finding 1 Gender***  ***Participants’ quotations:*** “Yes, usually women take care of the elderly family members, but some are not happy about it but they do it.  ***Authors’ interpretation:*** Anticipated acceptance: She described that it was expected of her family but also her community that she would take over this role. Traditional role: According to Miho, the responsibility for taking care of elderly family members was almost at all times assigned to the women when the time came. Historical adjustment: was a recurrent topic during the interview that the younger generation did not have the same respectful attitude toward elderly persons as their own parents probably had. | | |
| ***Finding 2 Reciprocity***  ***Participants’ quotations:*** “When I am not home, I can call my neighbors and they take care of my parents. We look after one another. ***Authors’ interpretation:*** Belonging. The important role of informal support that took place in a neighborhood where the elderly lived was also a frequent subject matter during the interview. It was observed that the use of “I” was immediately changed to “we” when a general opinion was expressed. Family resources. Concerns and worries related to ﬁnances were often expressed by Miho. Miho expressed frequently during the interview the importance of intergenerational contact. She stated that it was her observation that grandchildren were not spending as much time with their grandparents in Tokyo compared with the more traditional countryside. Adaptive creativity. Miho voiced conﬁdence when discussing the concrete opportunities of intergenerational relations. Throughout the narrative, the implication of Confucianism, strongly based on exchange and mutual beneﬁt or reciprocity, on familial expectations is present. | | |
| **Study**  **Authors ‘findings** | ***“All my life is one big nursing home”: Russian immigrant women in israel speak about double caregiver stress***  ***Remennick, 2002*** | |
| ***From abstract:*** Coupled with the challenges of resettlement, this double caregiver stress led to significant emotional and physical burnout. Exhaustion and tight time budgets led to health problems and poor self-care among these women. The informants’ social networks were mainly coethnic, and their coping tools drew on the Israeli–Russian community. The study concludes that, even in the relatively egalitarian Russian–Soviet gender system, women function as principal caregivers, often at the expense of other life goals. | | |
| ***Finding 1 Occupational and social downgrading***  ***Participants’ quotations:*** “None of us aspired to this kind of daily routine at work when we embarked on emigration.”  ***Authors’ interpretation:*** Functioning as caregivers for two generations, while giving them much power in the domestic realm, often hinders immigrant women from pursuing upward occupational mobility in the new society. | | |
| ***Finding 2 Family-related pressures***  ***Participants’ quotations:*** “Men have more to lose, and they are less ready for these losses—we should make things easier for them,”  ***Authors’ interpretation:*** The flow of support and instrumental help was often imbalanced or even one way—from the women “in the middle” to the rest of the extended family members. | | |
| ***Finding 3 Elder care***  ***Participants’ quotations:*** “you wouldn’t like to be done to yourself when you get old.”  ***Authors’ interpretation:*** In moral terms, placement was seen as a betrayal of a helpless parent. | | |
| ***Finding 4 Some outcomes of double caregiving***  ***Participants’ quotations:*** “ So my life is one big nursing home.”  ***Authors’ interpretation:*** Successful coping also depended on the amount of help they could summon from other able-bodied family members. Chronic role strain, aggravated by some physically demanding tasks, often resulted in various health problems, both somatic and psychological. | | |
| ***Finding 5 Social support: the role of “Russian” networks.***  ***Participants’ quotations:*** “My female friends, fellow immigrants, often prove more understanding and helpful than the family.”  ***Authors’ interpretation: I***n their daily struggles, my informants invariably sought, and found, support among their immigrant friends. | | |
| **Study**  **Authors’ findings** | ***Real men, real husbands: Caregiving and masculinities in later life***  ***Ribeiro et al., 2007*** | |
| ***From abstract:*** Through analytic methods based upon content analysis and open coding, authors found that when describing their gendered understanding of themselves participants evidenced several negotiations with the dominant masculine ideology in order to maintain their sense of masculinity and legitimate their presence in a feminine role. This was accomplished by reframing their definition of a man and reinforcing that of a husband and by retaining varying degrees of power over the caregiving relationship. The social visibility of the role within particular gendered community-based social networks was found to be important in shaping these older men's masculinities. | | |
| ***Finding 1 A (wo)man in charge ‘Man/husband/ caregiver’ ‘Power and the caregiving relationship’***  ***Participants’ quotations:*** “I am a man–woman. I am a man, as a man, [participant's emphasis] you see? I worked all my life, I was a very hardworking man… then I came home, and I became a woman, you see? I started to be a woman.” “It's harder for a woman to take care of a man; a man has more strength than a woman” “Well, I feel no shame. [?]” “She now totally depends on me, there's no one else here but me.”  ***Authors’ interpretation***: In the participants' description of their experience as caregivers, their perception of being in a domain that endangered their sense of masculinity was salient. Caregiving was defined as something “logical” and “natural” within a marital relationship and that interpretation eliminated any potential threats to our participants' masculinity. The balance of power between the caregiver and the care-receiver was also found to be present in the husbands' position of enhanced authority while the wives assumed a dependent position, which was more prominent in the situations of moderate and severe ailment. | | |
| ***Finding 2 Social visibility of care, Perceived social honour’ ‘Role's social legitimation’ ‘Against the mainstream’***  ***Participants’ quotations:*** “People see me doing things and say, ‘you're really one of those husbands… one of those husbands like all should be!”  ***Authors’ interpretation:*** Perceived social honour, as a source of self-esteem and self-worth for older male carers has been related to positive aspects of care), and, in being salient in the participants' description of their everyday life with caregiving tasks, such positive visibility seems to play an important role in their sense of masculinity as well. | | |
| ***Finding 3 Contemporary notions of masculinity***  ***Participants’ quotations:*** “Thirty years ago it would be shameful for a man to do certain things.”  ***Authors’ interpretation:*** A very few participants, all belonging to a specific subgroup that had higher education and presented the higher incomes of the sample, had a particular understanding of masculinity. They personally defined caregiving as something normative for any person and not dependent of any gender ideology or kin relationship. In their opinion, caregiving within a marital relationship was something broadly accepted nowadays. | | |
| **Study**  **Authors’ findings** | **Sighs, smiles, and worried glances: How the body reveals women caregivers' lived experiences of care to older adults**  **Silverman, 2013** | |
| ***From abstract:*** The data revealed a caregiver habitus characterized by the performance of emotional labor. Performance was visible through dissonance between the caregivers' verbal and nonverbal expressions and pointed to a high degree of emotion and body management. | | |
| ***Finding 1 The caregiver habitus***  ***Participants’ observation:*** Bonnie is emptying dishes from the dishwasher. She looks up, a glass in her hand. She whispers quietly, “You never know. From moment to moment.”  ***Authors’ interpretation:*** The common dispositions among the caregivers indicate that when women become caregivers, they acquire a common code of socially imprinted, gendered expectations that are learned through the implicit and explicit messages of their social emplacement — watching others, interpreting statements from healthcare practitioners, and imagining what it means to be a caregiver. | | |
| ***Finding 2 Becoming versus performing the caregiver habitus***  ***Participants’ observation:*** She then turns to face the bathroom and calls to Molly in a cheerful voice, “You ok there Mom?”  ***Authors’ interpretation:*** The data illustrated that the caregivers performed on the front stage, or the public zone, as well as the backstage, where the performance is prepared. | | |
| ***Finding 3 Emotional labour***  ***Participants’ observation:*** She says “NOPE” and then ﬁnishes pulling down the diaper.  ***Authors’ interpretation:*** If performance was revealed through dissonance between the caregivers' verbal and nonverbal expressions, at the heart of this dissonance was the attempt to accomplish the emotional labor necessary for caregiving. | | |
| ***Finding 4 Intersubjectivity and relational boundaries***  ***Participants’ observation:*** She yells to him “WHY ARE YOU THROWING OUT MY BOX?” Eli opens his eyes and says, “What box?”  ***Authors’ interpretation:*** Eruptions of conflict challenged the performance of emotional labor, pushing forward a full range of sentiments. | | |
| ***Finding 5 Divestment in health capital***  ***Participants’ observation:*** Her right-hand rests on her forehead. “He used to take care of me, I was the sick one. So, it makes it very difﬁcult.” Susan raises her hands again to ﬁddle with her hair clip. “I was always known, as the sick one and all of a sudden he, he does this.”  ***Authors’ interpretation:*** The data demonstrated multiple examples of the women divesting in their health, or depriving themselves of their bodily or health needs, confirming in part what research has shown about the prevalent self-neglect or negative health behaviors among caregivers. Despite the underlying moral reasons, the caregivers' divestment in their own health nonetheless led to resentment. | | |
| **Study**  **Authors’ findings** | **Family Caregivers of Impoverished Mexican American Elderly Women: The Perceived Impact of Adult Day Care Centers**  ***Valadez et al., 2005*** | |
| ***From abstract:*** Emergent themes and categories included (a) caretaking and gender differences, (b) resistance to nursing homes, (c) negative perceptions of White caregivers, (d) perceived positive effect of ADCs on the elderly parent’s physical and emotional (depression) states and the elderly parent–caregiver relationship, and (d) the negative effects of stress associated with caregiving. | | |
| ***Finding 1 Caretaking and Gender differences***  ***Participants’ quotations:*** “Yeah, I just think it is the way that my parents raised us.”  ***Authors’ interpretation:*** FMPs reported that women were the predominant caregivers for the elderly in the Mexican American culture. | | |
| ***Finding 2 Resistance to Nursing Homes***  ***Participants’ quotations:*** “So, if that person gave you love while you were growing up, how could you repay them by putting them in a place where there is no love?”  ***Authors’ interpretation:*** The negative sentiment toward nursing homes tended to cluster around these facilities’ reputation for abuse. | | |
| ***Finding 3 Perceptions of White Caretakers***  ***Participants’ quotations:*** “Look, I don’t have anything against White people…We Mexicans do not do that.”  ***Authors’ interpretation:*** The responses were consistent in the belief that Whites did not care as much about their elderly relatives | | |
| ***Finding 4 Perceived Emotional State and Impact of ADC on Elderly Relative Depression Health changesRelationship between the FMP and the elderly relative.***  ***Participants’ quotations:*** “Before she came to the center, she was always getting sick and depressed.”  ***Authors’ interpretation:*** According to FMPs, isolation and lack of social stimulation were two of the main contributors to their elderly relative’s depression. | | |
| ***Finding 5 Caregiver Burden and Impact of Adult Day Care Center Services Worry and guilt Time for family.***  ***Participants’ quotations:*** “The ADC has helped a great deal. I leave her off and I feel relieved.”  ***Authors’ interpretation:*** Two dimensions of worry and guilt emerged from the interviews. The first was related to FMPs’ natural preoccupation with their elderly relatives’ well-being while they were away at work. The second dimension of worry and guilt had a stronger reflective component. | | |
