# Supplementary material for: Gendered experiences of providing informal care for older people: a systematic review and thematic synthesis
Source: BMC Health Serv Res. 2021 Jul 23;21:730. doi: 10.1186/s12913-021-06736-2 (PMC8306003; doi:10.1186/s12913-021-06736-2)
Supplement: Supplementary file 3 — Additional file 3. [file 12913_2021_6736_MOESM3_ESM.docx]

**Additional Material 3.** *Demographic characteristics of the participants of the included studies*

| **Study** | **Number and Gender** | **Age** | **Country of Study** | **Occupation or Class** | **Relationship to the Care Receiver** |
| --- | --- | --- | --- | --- | --- |
| ***Black et al., 2008*** | 4 Male | 80+ | USA | Retired | Spouse/Partner |
| ***Cahill, 2000*** | 26 Male | 70-79 | Australia | Professional, Managerial, Skilled, Unskilled, Retired | Spouse/Partner |
| ***Calasanti and Bowen, 2006*** | 9 Male/ 13 Female | 53-78 | USA | Middle Class | Spouse/Partner |
| ***Calasanti and King, 2007*** | 9 Male | 65-83 | USA | Upper, Middle, Working Class | Spouse/Partner |
| ***Drummond et al. 2013*** | 6 Female | 60-80 | Canada | Unassigned | Spouse/Partner |
| ***Eriksson et al., 2013*** | 12 Female | 60-80 | Sweden | Unassigned | Spouse/Partner |
| ***Flores et al., 2009*** | 1 Female | 52 | USA | Working Class | Daugther |
| ***Hashizume, 2010*** | 11 Female | 39-52 | Japan | Employed, Self Empooyed | Daugther/ Daughter-In-Law |
| **Hayes et al., 2009** | 13 Male/ 15 Female | 50-80+ | USA | Unassigned | Spouse/Partner |
| ***Hayes et al., 2010*** | 13 Male/ 15 Female | 50-80+ | USA | Unassigned | Spouse/Partner |
| ***Hepburn et al., 2002*** | 39 Male/ 93 Female | 73 mean age | USA | Not Employed (Mainly), Part Time, Full Time | Spouse/Partner |
| ***Holroyd, 2005*** | 20 Female | 65-91 | China | Not Employed/ Previously Employed as Maids | Spouse/Partner |
| **Jones et al., 2002** | 41 Female | 38-68 | USA | Housewives, Work in Health and No Health-Related Field | Daugther/ Daughter-In-Law |
| **Kluczyńska, 2015** | 10 Male | 64-90 | Poland | Various Occupational Backgrounds | Spouse/Partner |
| ***Kramer, 2005*** | 36 Female | 42-86 | USA | Unassigned | Spouse, Parent, Sibling, Other Relative |
| ***Mendez et al., 2008*** | 41 Female | 19-83 | Mexico | Unemployed, Part Time, Full Time | Daughter, Granddaughter, Daughter-In-Law, Spouse |
| ***Paillard-Borg and Stromberg, 2014*** | 1 Female | 40+ | Japan | Educated as a Teacher | Daugther |
| ***Remennick, 2002*** | 20 Female | 49 Mean Age | Israel | Nursing, Public Social Services | Daughter |
| ***Ribeiro et al., 2007*** | 53 Male | 65-89 | Portugal | Retired, Part Time Job, Low Education | Spouse/Partner |
| **Silverman, 2013** | 5 Female | 48-63 | Canada | Unassigned | Spouse/Partner/ Daughter |
| ***Valadez et al., 2005*** | 1 Male/ 14 Female | 24-55 | USA | Full Time Employed | Son/Daughter |
